# Supplementary material for: Concentrated solar CO2 reduction in H2O vapour with >1% energy conversion efficiency
Source: Nat Commun. 2024 Jun 1;15:4675. doi: 10.1038/s41467-024-49003-8 (PMC11144235; doi:10.1038/s41467-024-49003-8)
Supplement: Supplementary file 1 — Supplementary information [file 41467_2024_49003_MOESM1_ESM.pdf]

Supplementary Information for

# Concentrated solar $CO_2$ reduction in $H_2O$ vapour with >1% energy conversion efficiency

Yuqi Ren [1], Yiwei Fu [2], Naixu Li [1,*], Changjun You [1], Jie Huang [2], Kai Huang [1], Zhenkun Sun [3], Jiancheng Zhou [1], Yitao Si [1], Yuanhao Zhu [1], Wenshuai Chen [4], Lunbo Duan [3,*], Maochang Liu [2,*]

[1] *School of Chemistry and Chemical Engineering, Southeast University, No.2 Dongnandaxue Road, Nanjing 211189, Jiangsu, P.R. China*

[2] *International Research Center for Renewable Energy, State Key Laboratory of Multiphase Flow in Power Engineering, Xi'an Jiaotong University, No.28 Xianning West Road, Xi'an, Shaanxi 710049, P.R. China*

[3] *Key Laboratory of Energy Thermal Conversion and Control of Ministry of Education, School of Energy and Environment, No.2 Dongnandaxue Road, Nanjing 210096, Jiangsu, P.R. China*

[4] *Key Laboratory of Bio-Based Material Science and Technology, Ministry of Education, Northeast Forestry University, No. 26 Hexing Road, Harbin 150040, P.R. China*

***** Corresponding Authors.**

*E-mail: naixuli@seu.edu.cn (N. L.), duanlunbo@seu.edu.cn (L. D.), maochangliu@mail.xjtu.edu.cn (M. L.)*

***These authors contributed equally:*** *Yuqi Ren, Yiwei Fu*

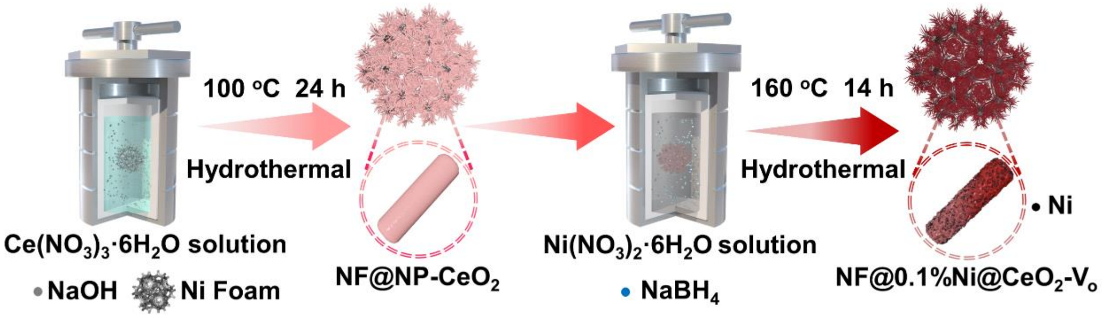


**Supplementary Fig. 1 |** Fabrication process of the monolithic NF@0.1%Ni@$CeO_2$-$V_o$ catalyst.

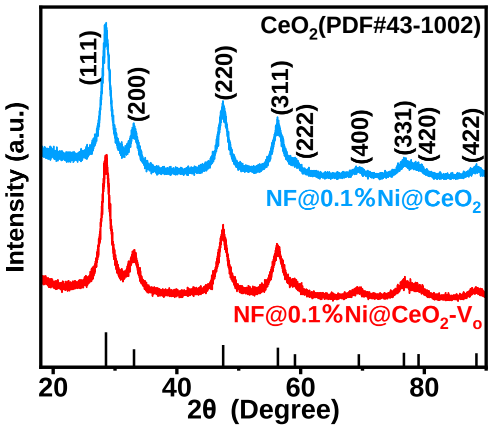


**Supplementary Fig. 2 |** XRD patterns of NF@0.1%Ni@$CeO_2$ and NF@0.1%Ni@$CeO_2$-$V_o$ catalysts.

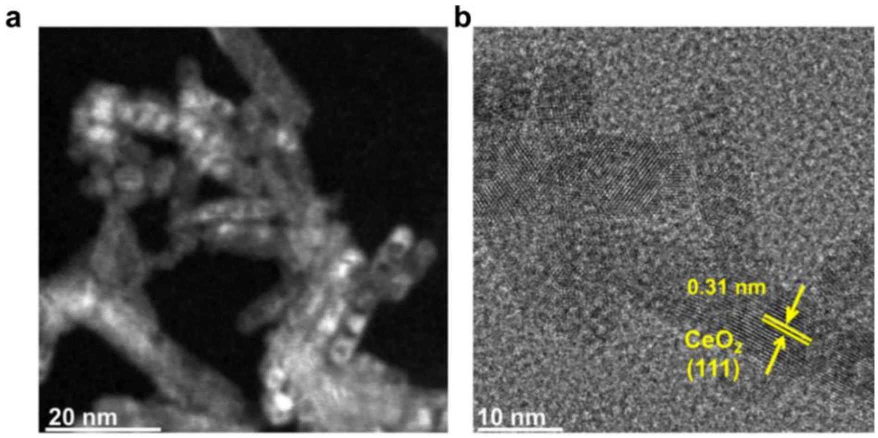


**Supplementary Fig. 3 | a, b** TEM images of NF@0.1%Ni@$CeO_2$ catalyst (**a**), and HRTEM images of the NF@0.1%Ni@$CeO_2$ catalyst (**b**).

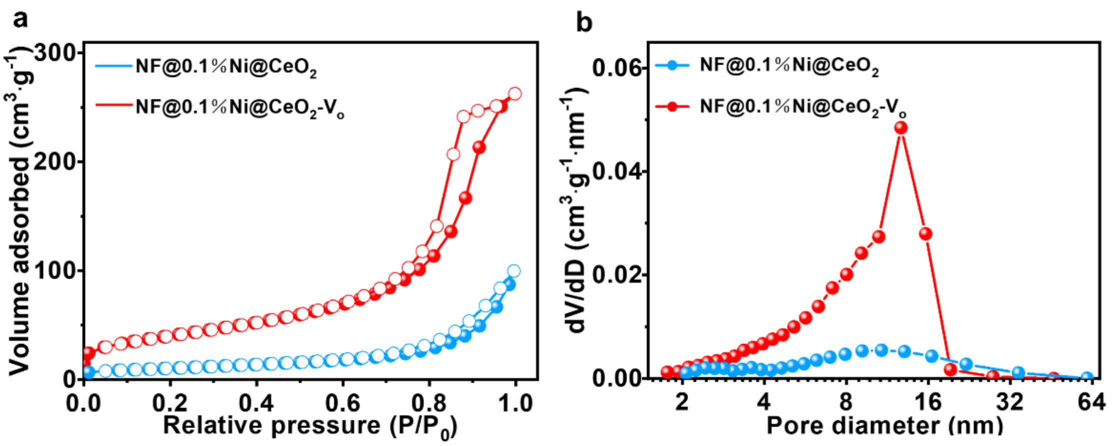


**Supplementary Fig. 4 | a, b** BET (**a**) corresponding pore size distribution curves (**b**) of NF@0.1%Ni@$CeO_2$ and NF@0.1%Ni@$CeO_2$-$V_o$ catalysts.

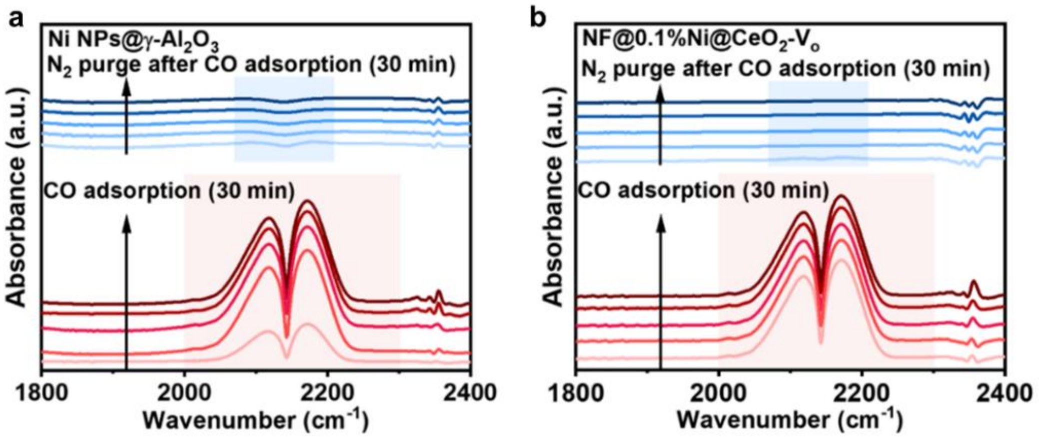


**Supplementary Fig. 5 | a, b** In-situ CO-DRIFT images of Ni NPs@γ-$Al_2O_3$ catalyst (**a**), and NF@0.1%Ni@$CeO_2$-$V_o$ catalyst (**b**).

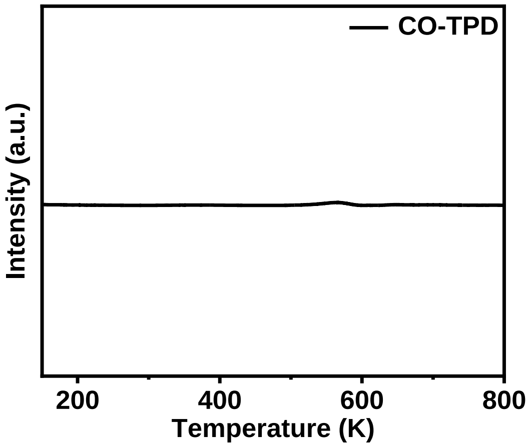


**Supplementary Fig. 6 |** CO-TPD images of NF@0.1%Ni@$CeO_2$-$V_o$.

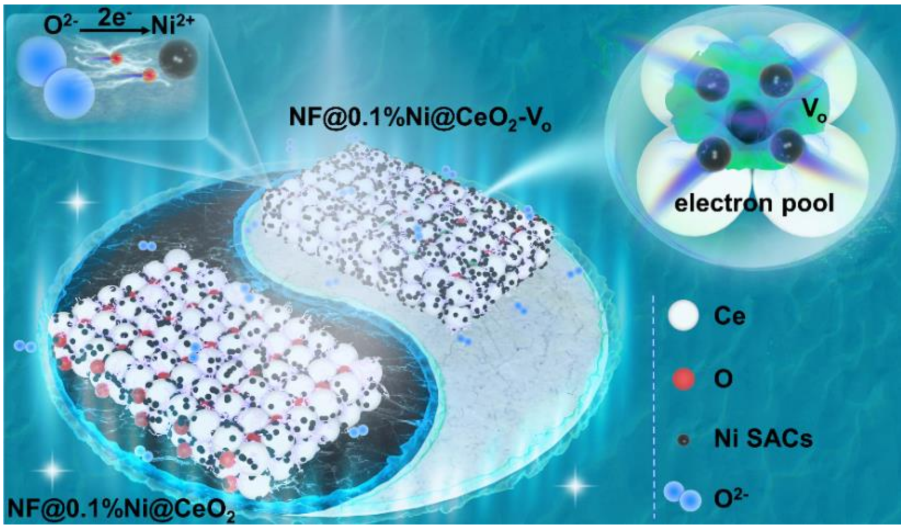


**Supplementary Fig. 7 |** Valence change diagram for Ni in the presence and absence of $V_o$ for NF@0.1%Ni@$CeO_2$ and NF@0.1%Ni@$CeO_2$-$V_o$ catalysts.

**Supplementary Discussion:** When the $O^{2-}$ is reduced to a $V_o$ by supplying electrons to Ni, the absence of the $O^{2-}$ provides a localized position for the $V_o$ to accept electrons and form an "electron pool" with the surrounding metal ions. The metal ions around this position form covalent bonds with the electrons in the $V_o$ through their 3*d* orbitals, allowing the electrons to cross the 3*d* orbitals of the inverted metal ions from the $V_o$. In this process, there is an overlap between the atomic orbitals of the metal ion and the molecular orbitals of the $V_o$, so that their electron densities can be exchanged and the 3*d* orbitals of the metal ion usually have unfilled or partially filled electronic states and can therefore receive electrons from the $V_o$.

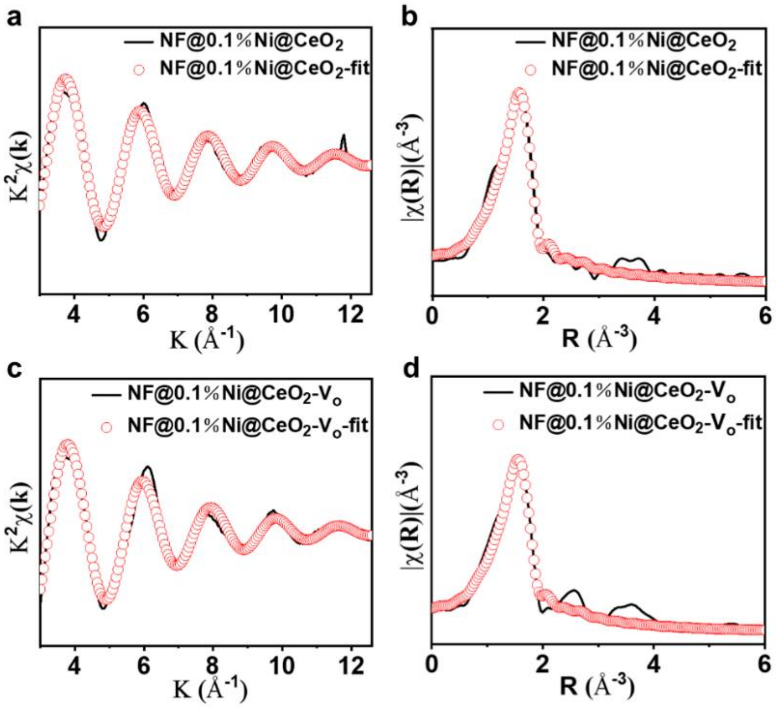


**Supplementary Fig. 8 | a, c** EXAFS fitting curve for the NF@0.1%Ni@$CeO_2$ (**a**) and NF@0.1%Ni@$CeO_2$-$V_o$ (**c**) catalysts in the region of 1.0-2.0 Å, shown in $k^2$ weighted R-space. **b, d** Ni K-edge EXAFS fit (line) for the NF@0.1%Ni@$CeO_2$ (**b**) and NF@0.1%Ni@$CeO_2$-$V_o$ (**d**), shown in $k^2$ weighted k-space.

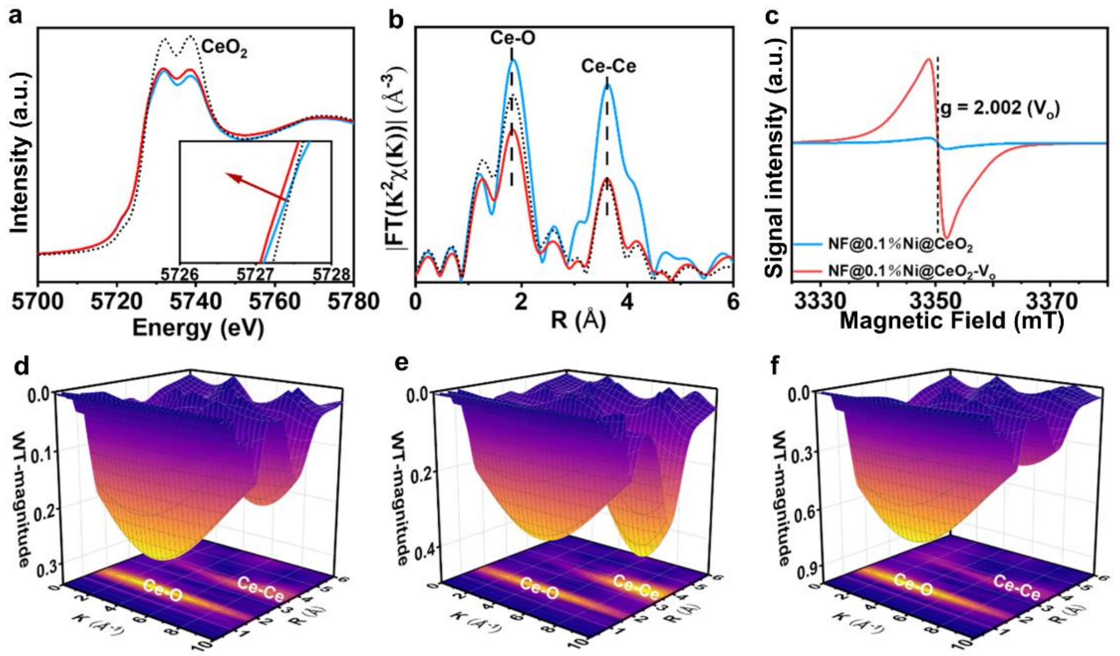


**Supplementary Fig. 9 | a** Normalized X-ray absorption near-edge spectra at the Ce $L_{III}$-edge. **b** $k^2$-weighted Fourier transform extended X-ray absorption fine structure spectra (EXAFS) in r-space (Blue for the NF@0.1%Ni@$CeO_2$ catalyst, red for the NF@0.1%Ni@$CeO_2$-$V_o$ catalyst). **c** EPR spectra of the NF@0.1%Ni@$CeO_2$ and the NF@0.1%Ni@$CeO_2$-$V_o$ catalyst; **d-f** Wavelet Transformation for the $k^2$-weighted EXAFS signal of $CeO_2$ reference (**d**), the NF@0.1%Ni@$CeO_2$ (**e**) and the NF@0.1%Ni@$CeO_2$-$V_o$ catalyst (**f**).

**Supplementary Discussion:** The electronic and coordination structures of the Ce samples were further supported by XAS techniques. Supplementary Fig. 9a shows the X-ray absorption near edge spectra (XANES) of the Ce $L_{III}$-edge of the NF@0.1%Ni@$CeO_2$-$V_o$ and NF@0.1%Ni@$CeO_2$ catalyst. The $E_0$ value of the NF@0.1%Ni@$CeO_2$-$V_o$ sample is lower than that of $CeO_2$, which suggests that the Ce atom has a positive charge of +3. On the other hand, the $E_0$ value of the NF@0.1%Ni@$CeO_2$ sample is comparable to that of $CeO_2$, indicating that the Ce atom has a positive charge of +4. The presence of oxygen vacancies can lead to a shift in the oxidation state of Ce from +4 to +3, which is consistent with the results obtained from the EPR analysis (Supplementary Fig. 9c). As shown in the Fourier-transformed $k^2$ weighted EXAFS spectra at the Ce $L_{III}$-edge (Supplementary Fig. 9b), in contrast to the reference samples of $CeO_2$, the Ce catalyst showed two major peaks, which could be

ascribed to Ce–O and Ce–Ce. The Ce–O and Ce–Ce scattering is further demonstrated by the wavelet transform (WT) technique, shown in Supplementary Figs. 9d-f.

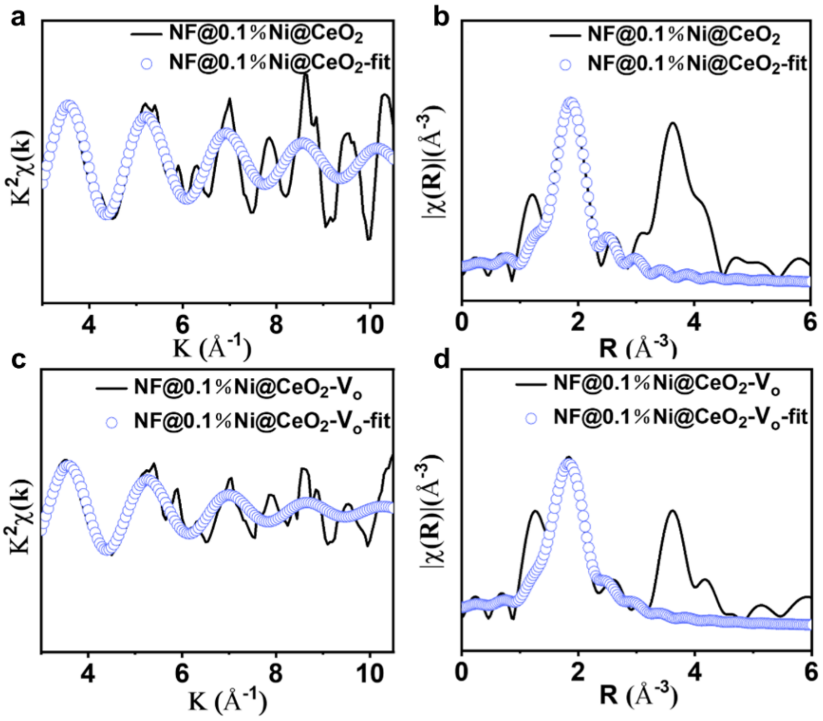


**Supplementary Fig. 10 | a, c** EXAFS fitting curve for the NF@0.1%Ni@$CeO_2$ (**a**) and NF@0.1%Ni@$CeO_2$-$V_o$ (**c**) catalysts in the region of 1.4-2.3 Å, shown in $k^2$ weighted R-space. **b, d** Ce $L_{III}$-edge EXAFS fit (line) for the NF@0.1%Ni@$CeO_2$ (**b**) and NF@0.1%Ni@$CeO_2$-$V_o$ (**d**), shown in $k^2$ weighted k-space.

**Supplementary Discussion:** The best-fitted EXAFS result revealed Ce–O at 2.28 Å with a CN of 6.1 for NF@0.1%Ni@$CeO_2$-$V_o$ sample, Ce–O at 2.31 Å with a CN of 7.1 for NF@0.1%Ni@$CeO_2$ catalyst, respectively (Supplementary Fig. 10 and Supplementary Table 4).

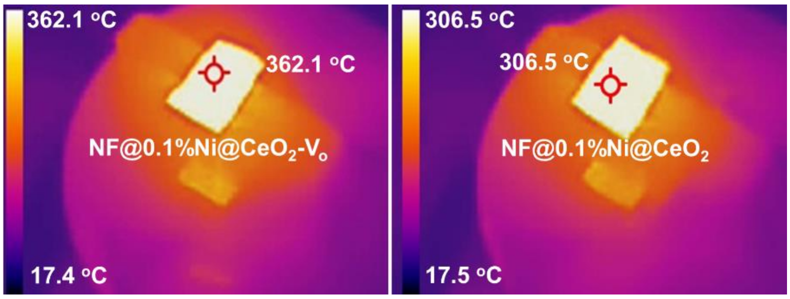


**Supplementary Fig. 11 |** Infrared images of the NF@0.1%Ni@$CeO_2$-$V_o$ and NF@0.1%Ni@$CeO_2$ catalysts under concentrated solar irradiation.

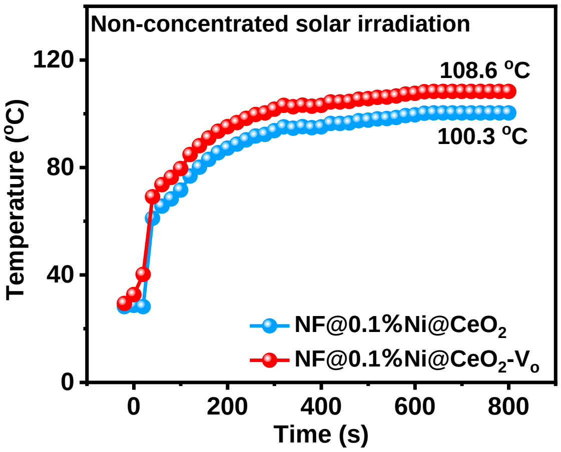


**Supplementary Fig. 12 |** Surface temperature of the NF@0.1%Ni@$CeO_2$ and NF@0.1%Ni@$CeO_2$-$V_o$ catalysts under non-concentrated solar irradiation measured by infrared imaging.

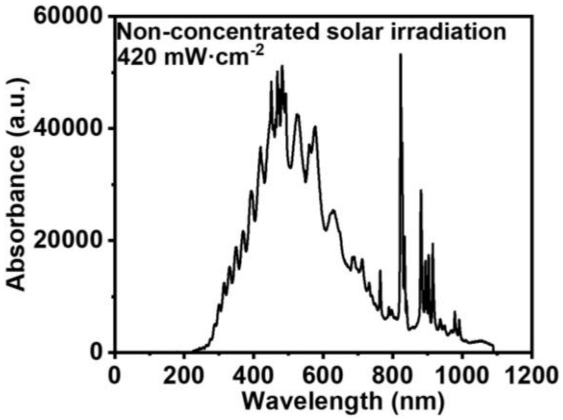


**Supplementary Fig. 13 |** Light source spectra of non-concentrated solar irradiation.

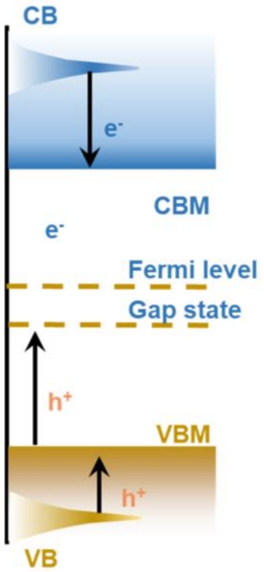


**Supplementary Fig. 14 |** Schematic diagram of photo-generated holes transferred to the gap state.

**Supplementary Discussion:** In the gap between VBM and CBM, gap states are not necessarily empty. For example, oxygen vacancies may introduce additional electrons, thereby increasing the Fermi level. At this point, the gap states are occupied by electrons. In our system, under the influence of nickel monomers and oxygen vacancies, the gap state lies below the Fermi level and is occupied under the non-excited condition. When electrons are excited from the VBM under light excitation, an empty state in the VBM is generated. Then the electrons of surface atoms or defects in the gap state can subsequently transition downwards to the VBM. This is analogous to photo-generated holes transitioning from the VBM to the gap states.

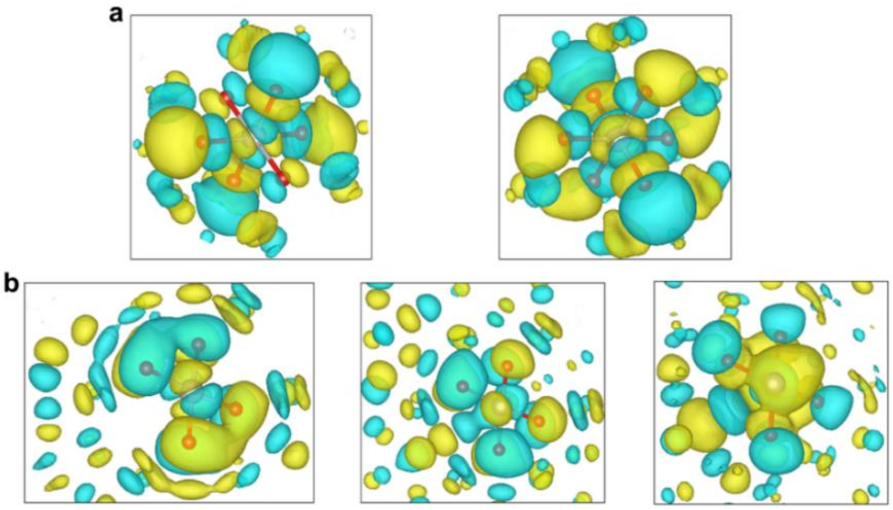


**Supplementary Fig. 15 | a, b** Wavefunction distribution of the bandgap states for NF@0.1%Ni@$CeO_2$ (**a**) and NF@0.1%Ni@$CeO_2$-$V_o$ (**b**).

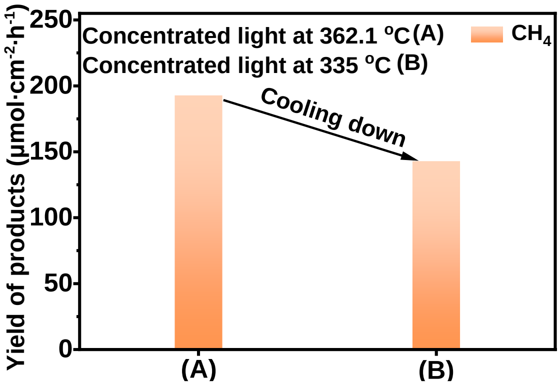


**Supplementary Fig. 16 |** The yield of $CH_4$ over the NF@0.1%Ni@$CeO_2$-$V_o$ catalyst under different conditions.

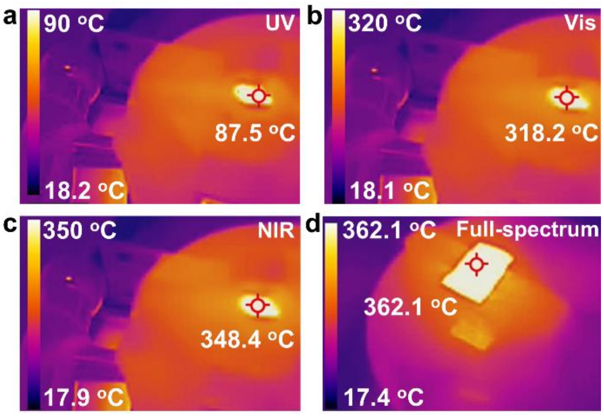


**Supplementary Fig. 17 | a-d** Temperatures of the NF@0.1%Ni@$CeO_2$-$V_o$ under UV (**a**), Vis (**b**), NIR (**c**) and full-spectrum (**d**) concentrated solar irradiation.

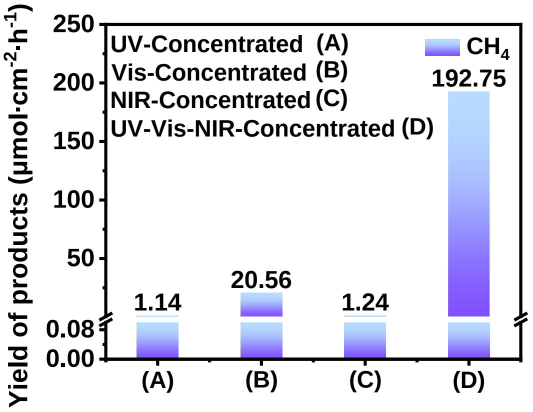


**Supplementary Fig. 18 |** The yield of products under different wavelength ranges of concentrated light.

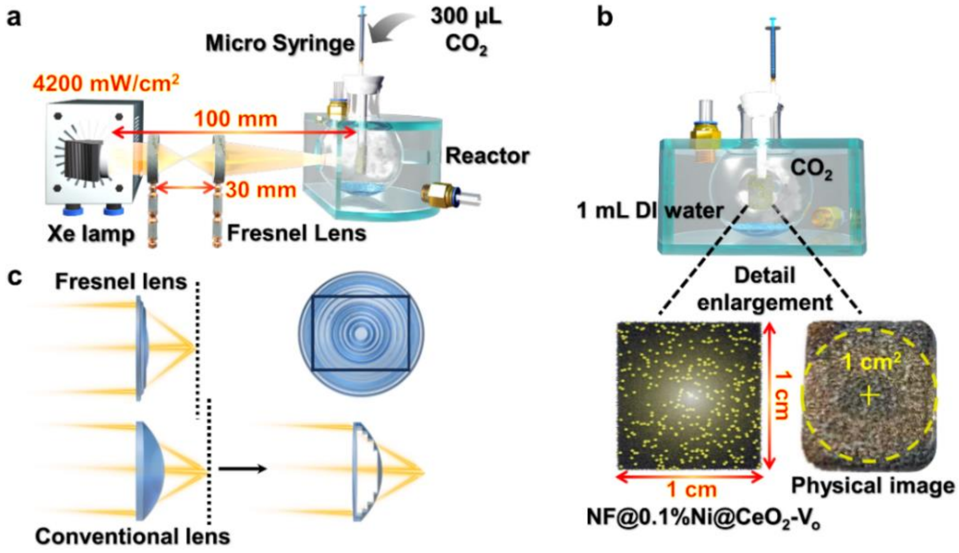


**Supplementary Fig. 19 | a, b** Schematic diagram of photo-thermal coupling reaction device (**a**) and detail enlargement diagram (**b**). **c** Differences between a conventional lens and a Fresnel lens.

**Supplementary Discussion:** Unlike traditional lenses, the Fresnel lens replaces the curved surface of an optical lens with concentric grooves, which act as independent refractive surfaces that bend parallel light rays to a common focal length closer than a traditional lens. The low thickness also reduces the absorption of light by the lens itself, thus allowing it to be manufactured from cost-effective materials such as plastic (Supplementary Fig. 19c).

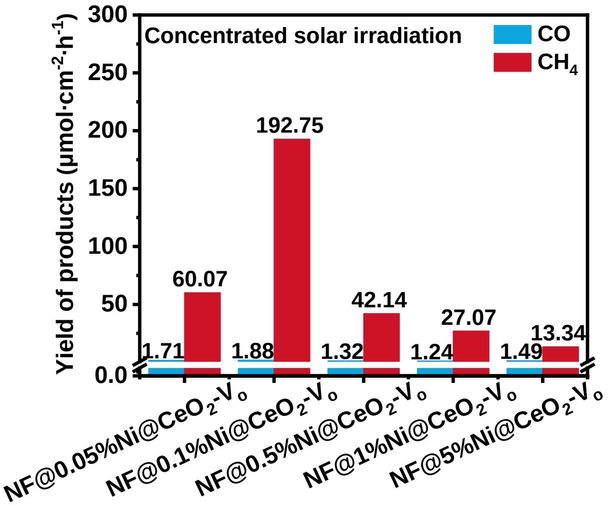


**Supplementary Fig. 20 |** Yield diagram for different amounts of Ni in the NF@0.1%Ni@$CeO_2$-$V_o$ catalyst under concentrated solar irradiation.

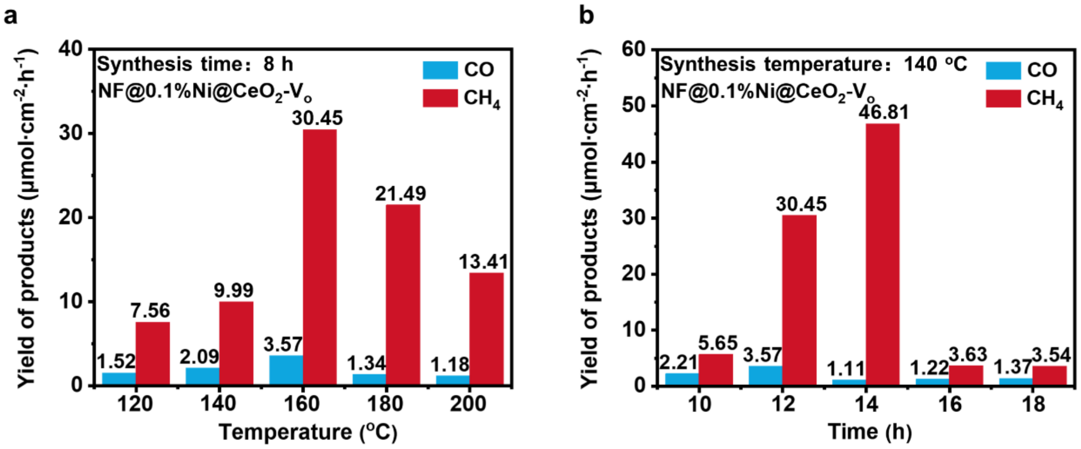


**Supplementary Fig. 21 | a** Yields of the NF@0.1%Ni@$CeO_2$-$V_o$ catalyst prepared at different temperatures under concentrated solar irradiation. **b** Yields of the NF@0.1%Ni@$CeO_2$-$V_o$ catalyst prepared at different synthesis times under concentrated solar irradiation.

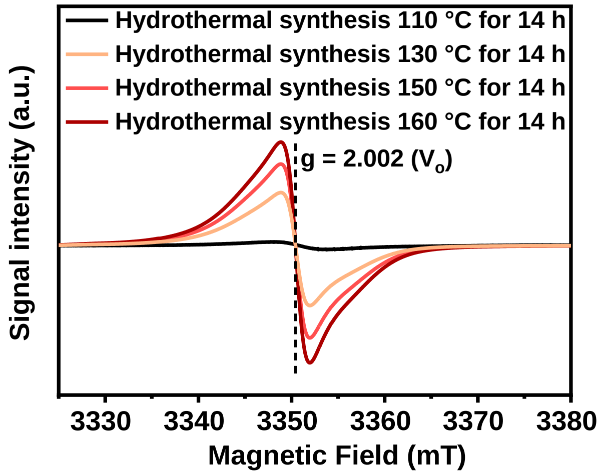


**Supplementary Fig. 22 |** EPR spectra of catalysts with different hydrothermal synthesis temperatures.

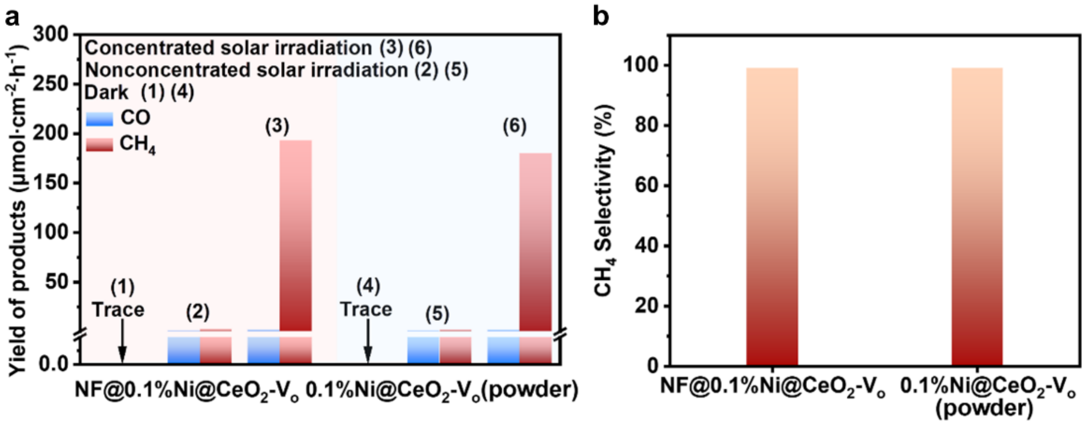


**Supplementary Fig. 23 | a, b** Photothermal catalytic $CH_4$ yields from NF@0.1%Ni@$CeO_2$-$V_o$ and 0.1%Ni@$CeO_2$-$V_o$ powder catalysts under different conditions (non-concentrated solar irradiation represents 420 mW/cm$^2$, and concentrated solar irradiation represents 4200 mW/cm$^2$) (**a**) and $CH_4$ selectivity of NF@0.1%Ni@$CeO_2$-$V_o$ and 0.1%Ni@$CeO_2$-$V_o$ powder catalysts (**b**).

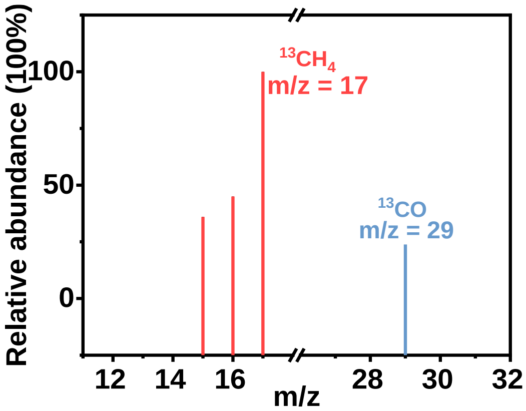


**Supplementary Fig. 24 |** $^{13}CO_2$ isotopic tracing experiments of NF@0.1%Ni@$CeO_2$-$V_o$.

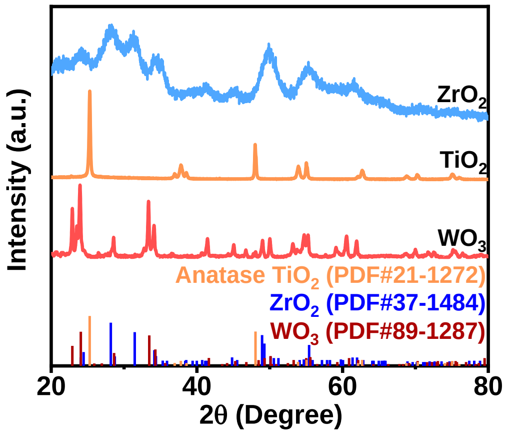


**Supplementary Fig. 25 |** XRD patterns of different catalysts.

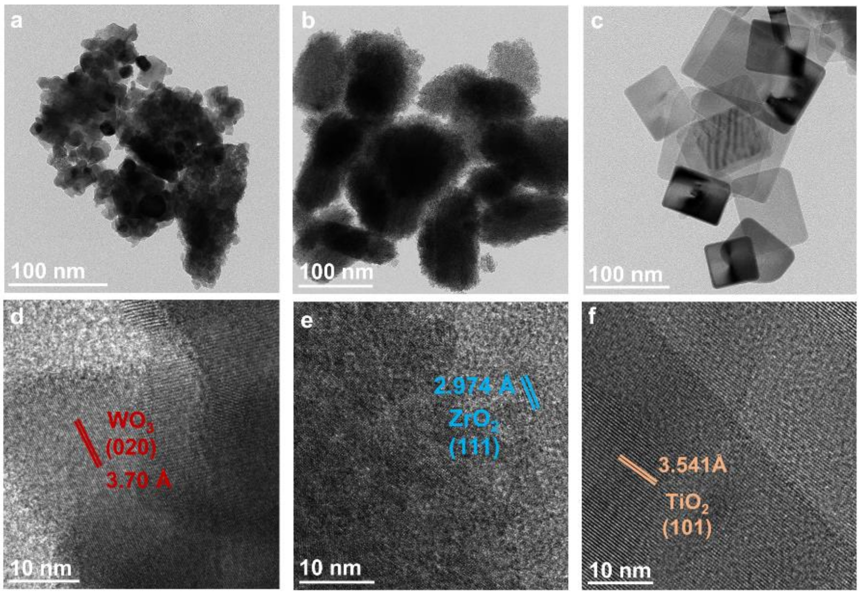


**Supplementary Fig. 26 | a, b, and c** TEM image of $WO_3$ (**a**), $ZrO_2$ (**b**), and $TiO_2$ (**c**) catalysts. **d, e, and f** HRTEM image of $WO_3$ (**d**), $ZrO_2$ (**e**), and $TiO_2$ (**f**) catalysts.

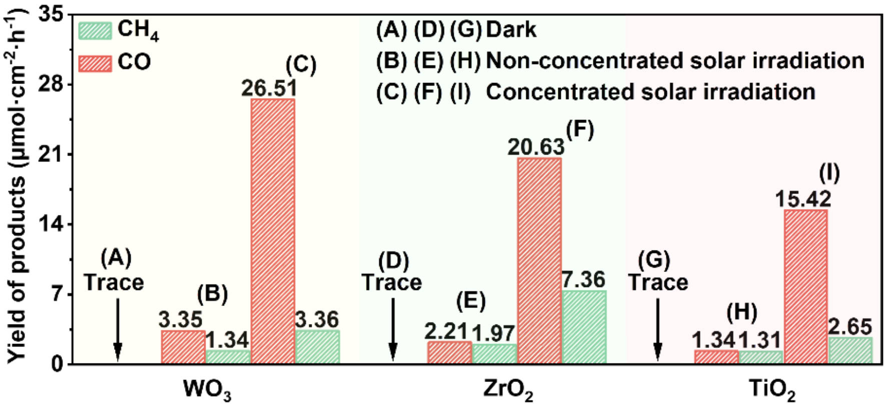


**Supplementary Fig. 27 |** Photothermal $CO_2$ reduction performance of $WO_3$, $ZrO_2$, and $TiO_2$ catalysts under different conditions.

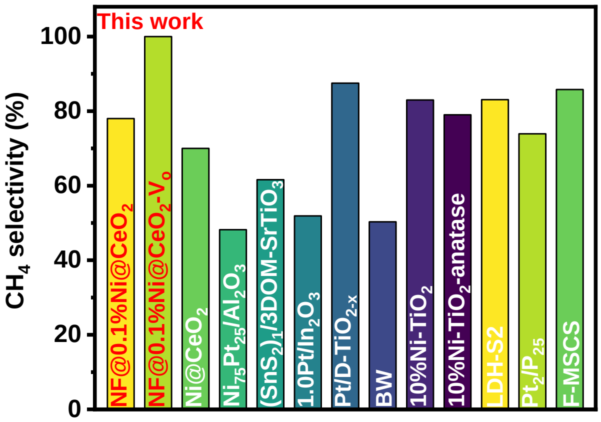


**Supplementary Fig. 28 |** Schematic comparison of $CH_4$ selectivity of the NF@0.1%Ni@$CeO_2$ and NF@0.1%Ni@$CeO_2$-$V_o$ catalysts with other catalysts under similar conditions.

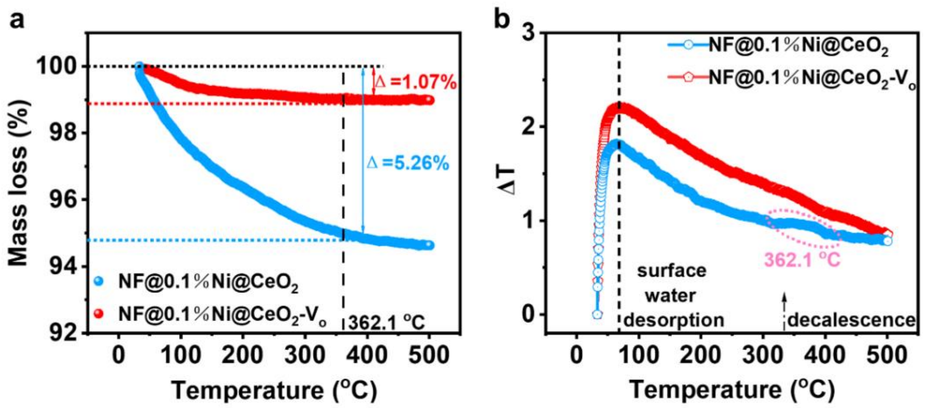


**Supplementary Fig. 29 | a, b** Differential thermal analysis (**a**) and thermogravimetric analysis curves (**b**) of the NF@0.1%Ni@$CeO_2$ and NF@0.1%Ni@$CeO_2$-$V_o$ catalysts.

**Supplementary Discussion:** The change in mass loss was 1.07% for catalyst NF@0.1%Ni@$CeO_2$-$V_o$ and 5.26% for catalyst NF@0.1%Ni@$CeO_2$ at a temperature of 361.2 °C. Meanwhile, catalyst NF@0.1%Ni@$CeO_2$ showed obvious heat absorption peaks, while catalyst NF@0.1%Ni@$CeO_2$-$V_o$ only had surface water desorption peaks. It indicates that catalyst NF@0.1%Ni@$CeO_2$-$V_o$ has high thermal stability under concentrated solar irradiation conditions.

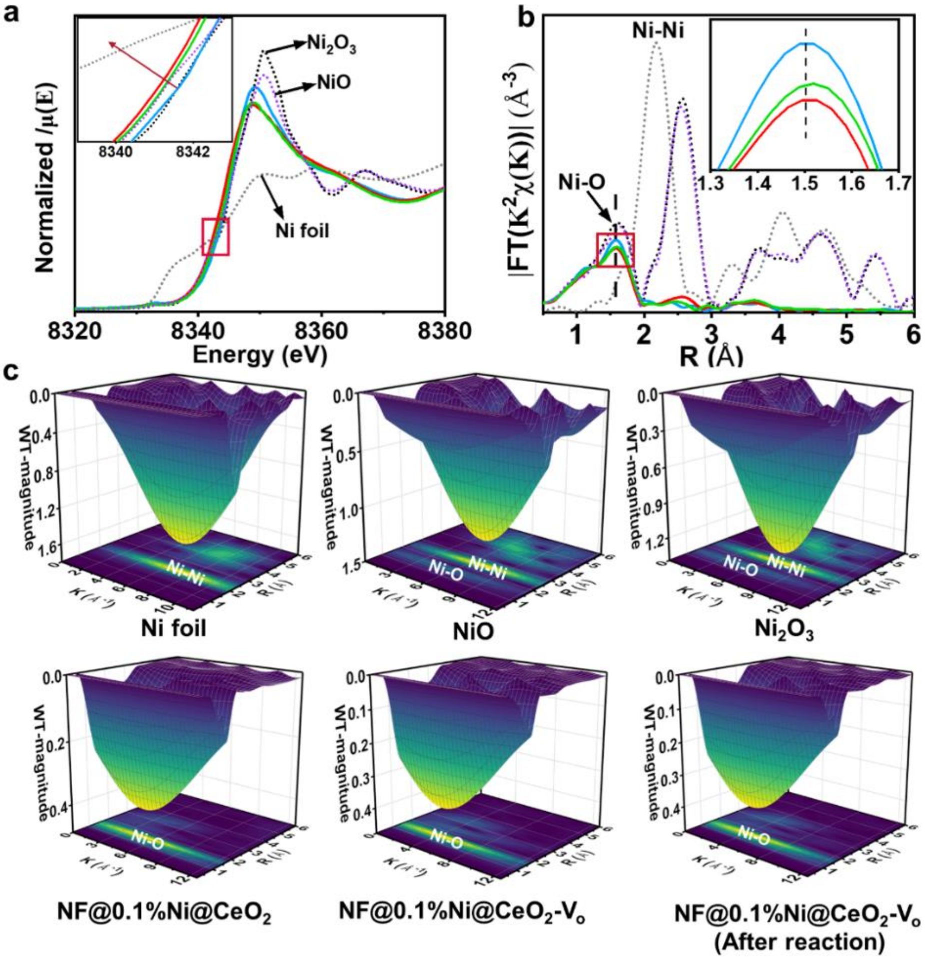


**Supplementary Fig. 30 | a** Normalized X-ray absorption near-edge spectra at the Ni K-edge. **b** $k^2$-weighted Fourier transform extended X-ray absorption fine structure spectra (EXAFS) in r-space (blue for the NF@0.1%Ni@$CeO_2$ catalyst, red for the NF@0.1%Ni@$CeO_2$-$V_o$ catalyst, and green for the NF@0.1%Ni@$CeO_2$-$V_o$ catalyst after reaction). **c** Wavelet Transformation for the $k^2$-weighted EXAFS signal of the Ni foil, NiO, $Ni_2O_3$, NF@0.1%Ni@$CeO_2$, NF@0.1%Ni@$CeO_2$-$V_o$, and NF@0.1%Ni@$CeO_2$-$V_o$ catalysts after reaction.

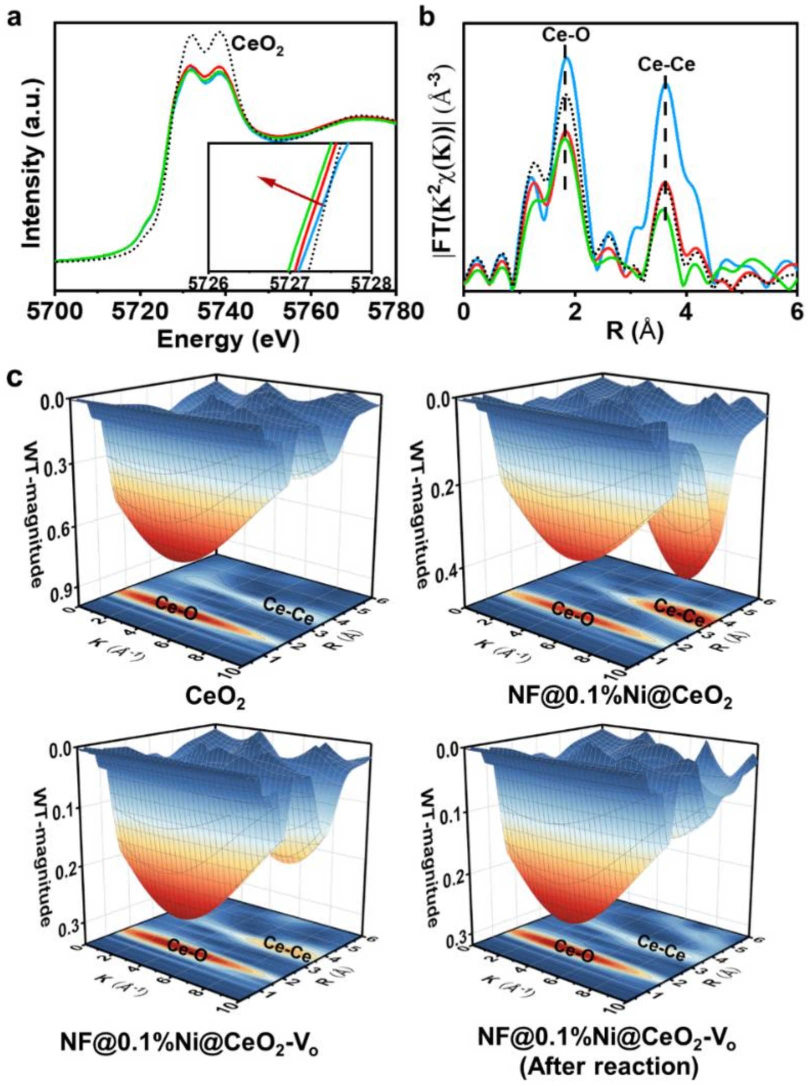


**Supplementary Fig. 31 | a** Normalized X-ray absorption near-edge spectra at the Ce $L_{III}$-edge. **b** $k^2$-weighted Fourier transform extended X-ray absorption fine structure spectra (EXAFS) in r-space (blue for the NF@0.1%Ni@$CeO_2$ catalyst, red for the NF@0.1%Ni@$CeO_2$-$V_o$ catalyst, and green for the NF@0.1%Ni@$CeO_2$-$V_o$ catalyst after reaction). **c** Wavelet Transformation for the $k^2$-weighted EXAFS signal of $CeO_2$ reference, NF@0.1%Ni@$CeO_2$, NF@0.1%Ni@$CeO_2$-$V_o$, and NF@0.1%Ni@$CeO_2$-$V_o$ catalysts after reaction.

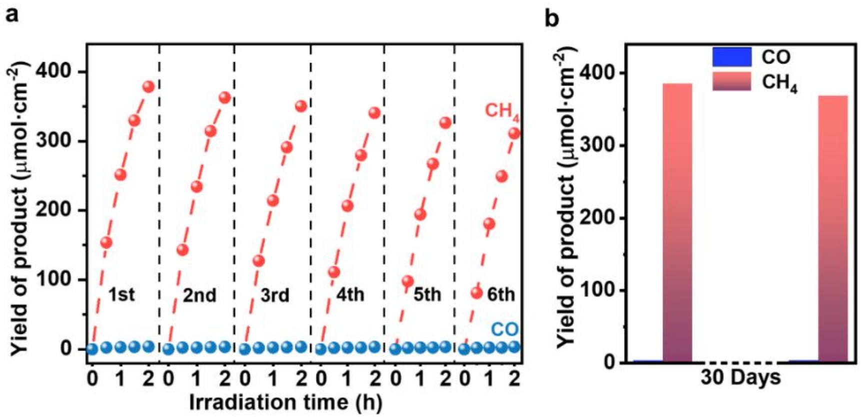


**Supplementary Fig. 32 | a** Recycling photothermal $CO_2$ tests of the NF@0.1%Ni@$CeO_2$-$V_o$ catalyst. **b** Comparison of photothermal $CO_2$ reduction yields of NF@0.1%Ni@$CeO_2$-$V_o$ catalyst kept under Ar atmosphere for 30 days.

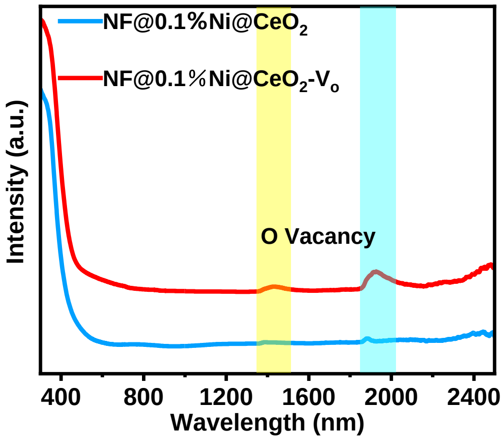


**Supplementary Fig. 33 |** UV-visible absorption spectra of the NF@0.1%Ni@$CeO_2$ and NF@0.1%Ni@$CeO_2$-$V_o$ catalysts.

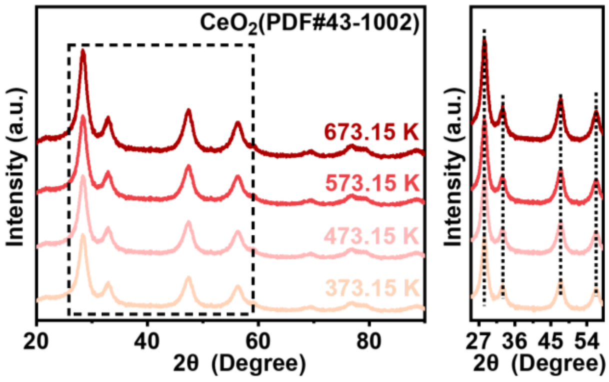


**Supplementary Fig. 34 |** In-situ XRD pattern of NF@0.1%Ni@$CeO_2$-$V_o$ under different temperatures.

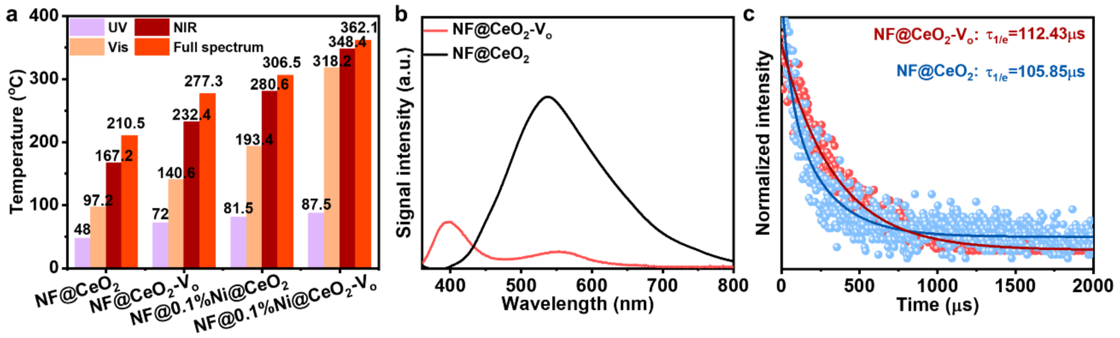


**Supplementary Fig. 35 | a** Surface temperature of different catalysts under concentrated light conditions. **b** PL spectra measured and for NF@CeO$_2$ and NF@CeO$_2$-V$_o$. **c** Time-resolved transient PL decay spectra.

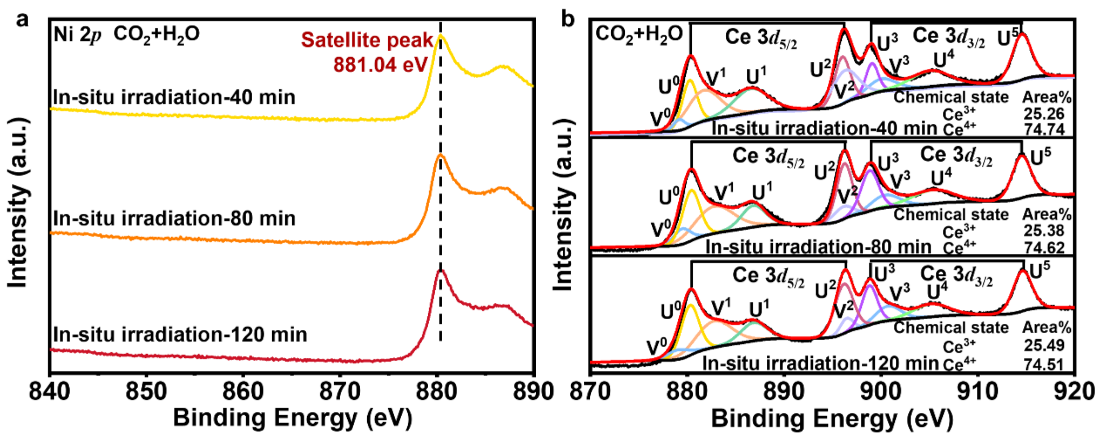


**Supplementary Fig. 36 | a** In situ Ni 2*p* XPS spectra for NF@0.1%Ni@CeO$_2$-V$_o$. **b** In situ Ce 3*d* XPS spectra for NF@0.1%Ni@CeO$_2$-V$_o$.

**Supplementary Discussion:** In-situ XPS spectra of the Ni 2*p* orbitals of the NF@0.1%Ni@CeO$_2$-V$_o$ catalyst after irradiation with the reactants $CO_2$ and $H_2O$ vapour for 120 min. However, the spectra only show the satellite peak at 881.04 eV. The signal intensity of Ni is very weak due to its low content (0.1%) (Supplementary Fig. 36a).

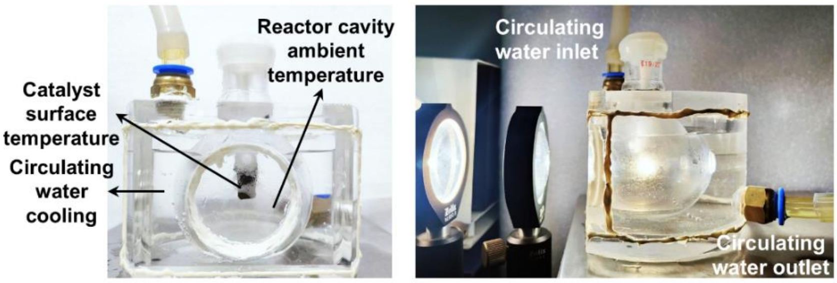


**Supplementary Fig. 37 |** Physical diagram of the catalytic process.

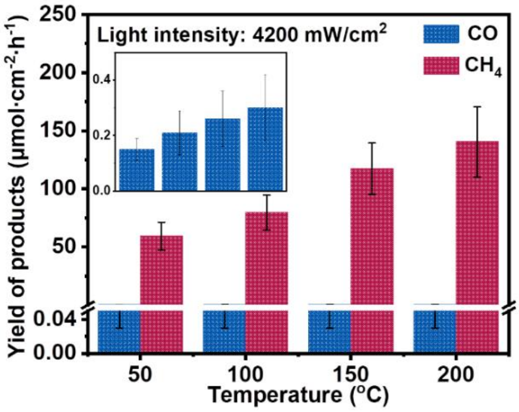


**Supplementary Fig. 38 |** $CO_2$ reduction performance of the NF@0.1%Ni@$CeO_2$-$V_o$ catalyst at different temperatures under concentrated solar irradiation conditions. The temperature refers to the ambient temperature of the reactor cavity during catalysis. Error bars are standard deviations were calculated from triple activity testing.

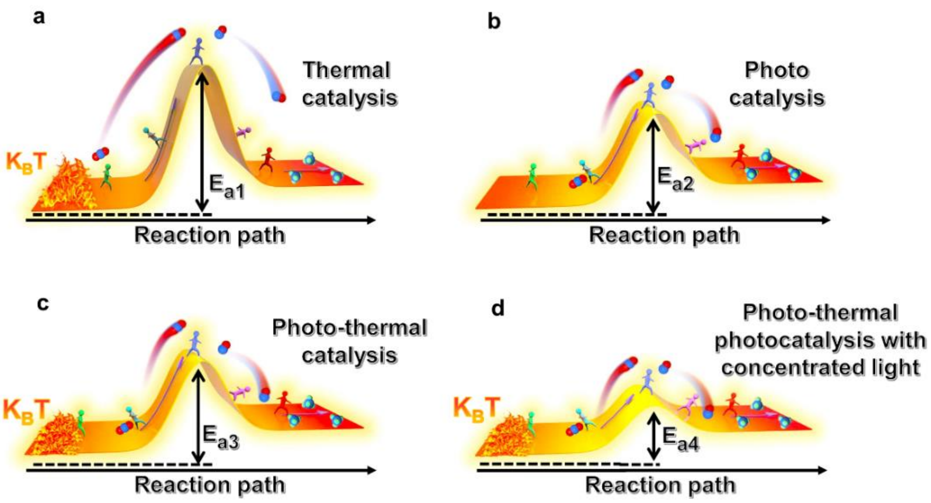


**Supplementary Fig. 39 |** Schematic diagram of the apparent activation energy of thermal catalysis (**a**), photocatalysis (**b**), photothermal catalysis (**c**), photo-thermal photocatalysis (d) with concentrated solar irradiation.

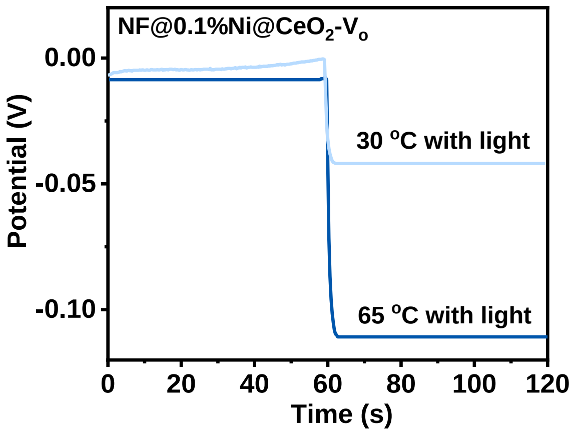


**Supplementary Fig. 40 |** Open circuit potential at different temperatures.

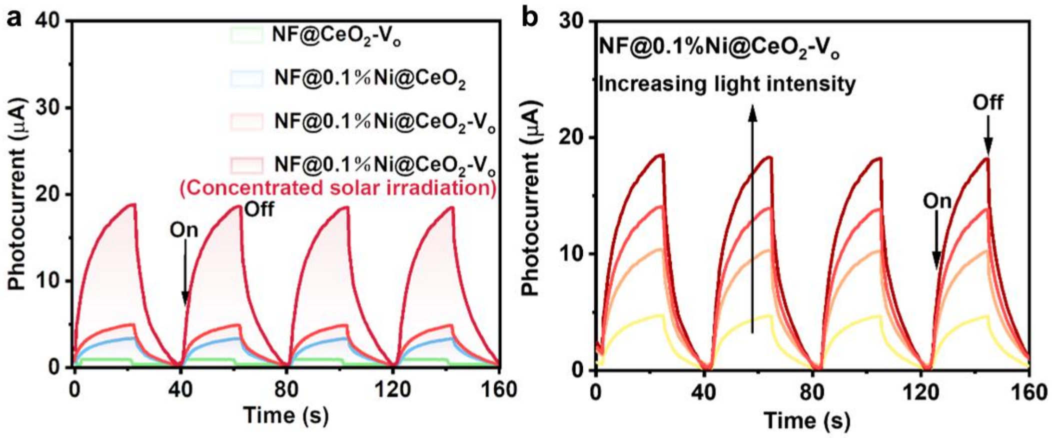


**Supplementary Fig. 41 | a** Transient photocurrent curves for different catalysts under concentrated solar irradiation. **b** Transient photocurrent density of NF@0.1%Ni@$CeO_2$-$V_o$ catalyst under different light intensities.

**Supplementary Discussion:** The photo-response of NF@0.1%Ni@$CeO_2$-$V_o$ was largest under concentrated solar irradiation conditions, suggesting that the high photo-electron flux density drives single-atom Ni and $V_o$ to further enhance the conductivity and carrier separation efficiency.

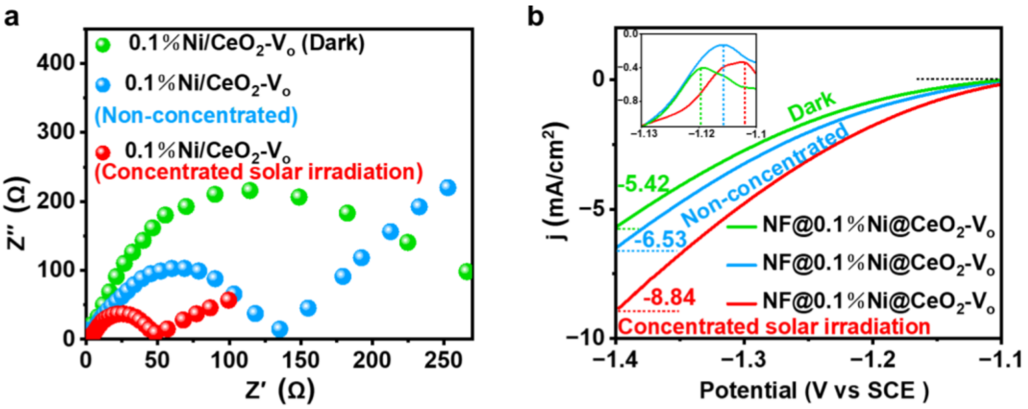


**Supplementary Fig. 42 | a** EIS Nyquist plots of NF@0.1%Ni@$CeO_2$-$V_o$ under different conditions. **b** LSV of NF@0.1%Ni@$CeO_2$-$V_o$ under different conditions.

**Supplementary Discussion:** The EIS of NF@0.1%Ni@$CeO_2$-$V_o$ was recorded at varying irradiation intensities, with the interfacial charge transfer being enhanced by the effective photon energy generated by the concentrated solar irradiation. Moreover, LSV tests were performed at different irradiation intensities, in which an electrical potential was applied to the catalyst electrode to drive the separation and transfer of photo-generated carriers.

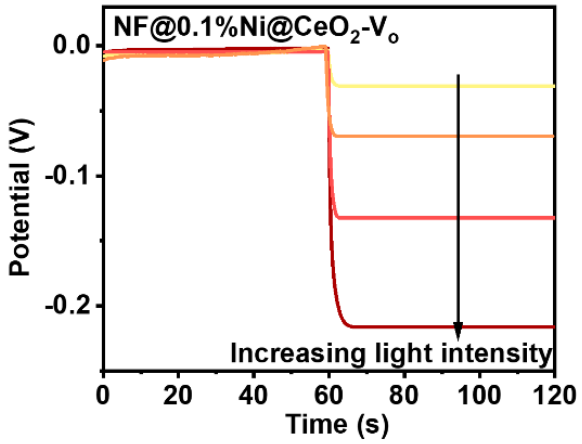


**Supplementary Fig. 43 |** Open circuit potential under different light intensities.

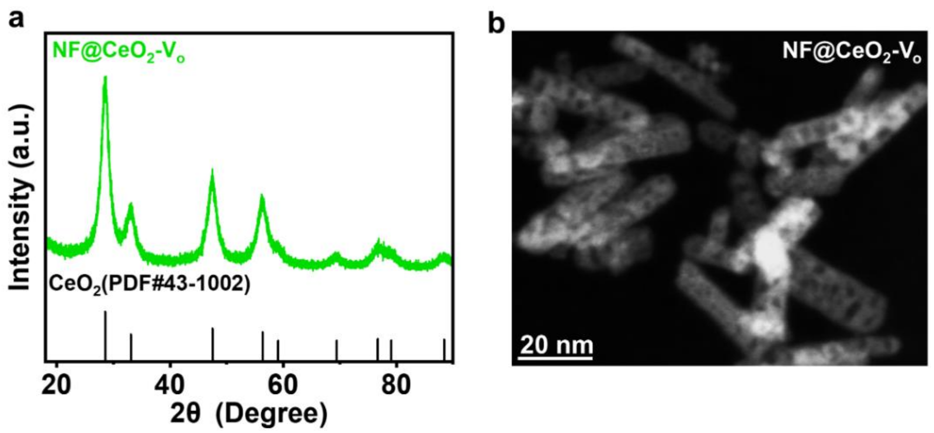


**Supplementary Fig. 44 | a, b** XRD pattern (**a**), and TEM image of the NF@$CeO_2$-$V_o$ catalyst (**b**).

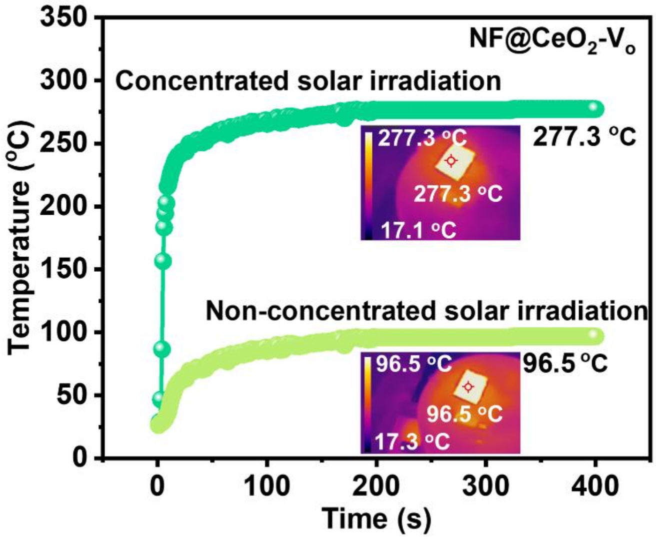


**Supplementary Fig. 45 |** Surface temperature of the NF@$CeO_2$-$V_o$ catalyst under non-concentrated solar irradiation and concentrated solar irradiation, insets are infrared imaging.

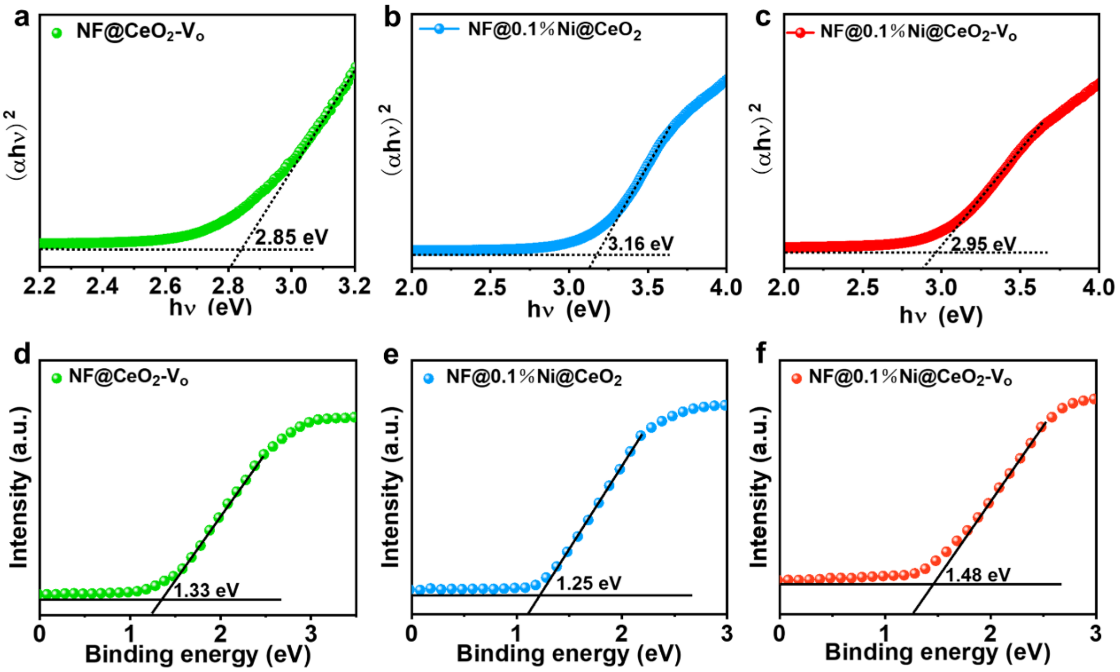


**Supplementary Fig. 46 | a-c** The Tauc plots of NF@$CeO_2$-$V_o$ (**a**), NF@0.1%Ni@$CeO_2$ (**b**), and NF@0.1%Ni@$CeO_2$-$V_o$ (**c**) catalysts. **d-f** XPS valence spectra of the NF@$CeO_2$-$V_o$ (**d**), NF@0.1%Ni@$CeO_2$ (**e**), and NF@0.1%Ni@$CeO_2$-$V_o$ (**f**) catalysts.

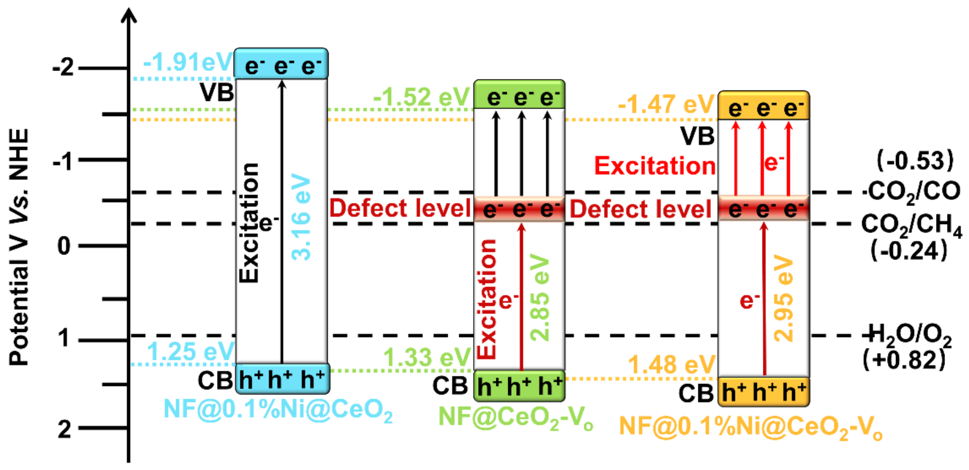


**Supplementary Fig. 47 |** Energy level diagram for the NF@$CeO_2$-$V_o$, NF@0.1%Ni@$CeO_2$, and NF@0.1%Ni@$CeO_2$-$V_o$ catalysts.

**Supplementary Discussion:** The synergistic effect of $V_o$ and Ni leads to a shift of CB towards a more positive potential, favoring the catalytic process of dissociation of $H_2O$ molecules.

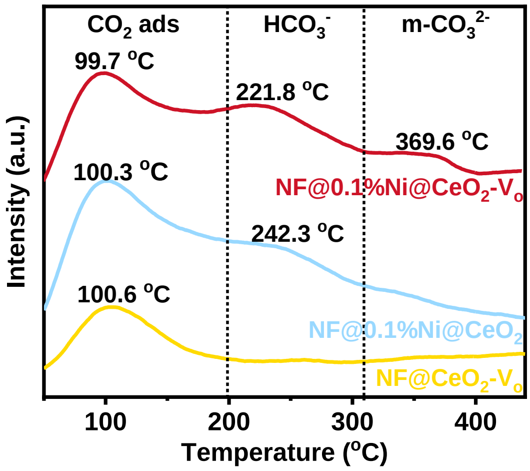


**Supplementary Fig. 48 |** $CO_2$-TPD spectra of the NF@$CeO_2$-$V_o$, NF@0.1%Ni@$CeO_2$, and NF@0.1%Ni@$CeO_2$-$V_o$ catalysts.

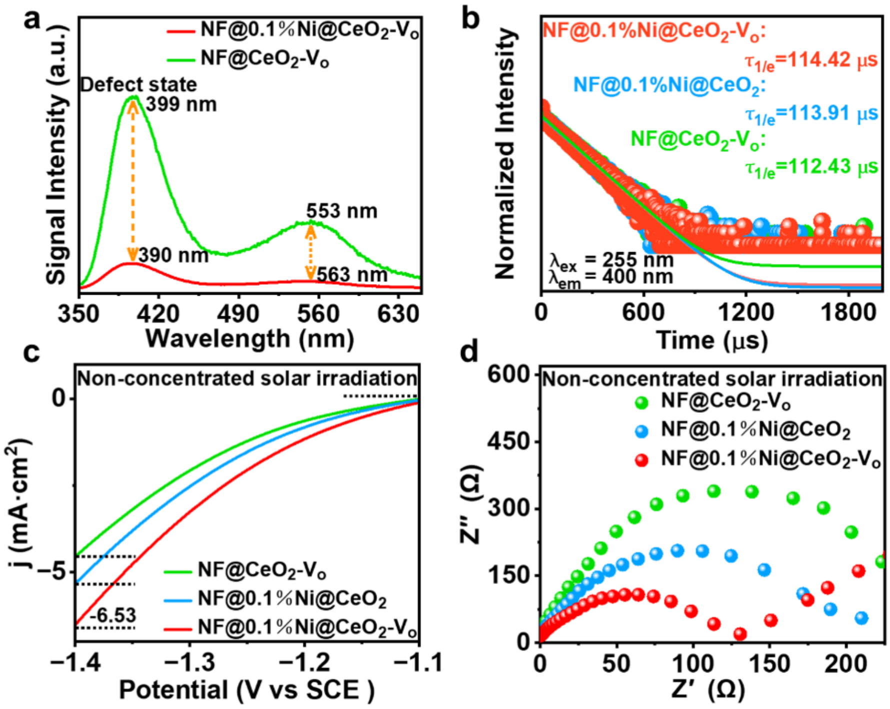


**Supplementary Fig. 49 | a** Steady-state PL spectra of NF:0.1%Ni@$CeO_2$-$V_o$ and NF@$CeO_2$-$V_o$. **b-d** Time-resolved transient PL decay spectra (**b**), LSV under non-concentrated solar irradiation (**c**), and EIS Nyquist plots under non-concentrated solar irradiation (**d**) of NF@$CeO_2$-$V_o$, NF@0.1%Ni@$CeO_2$ and NF@0.1%Ni@$CeO_2$-$V_o$.

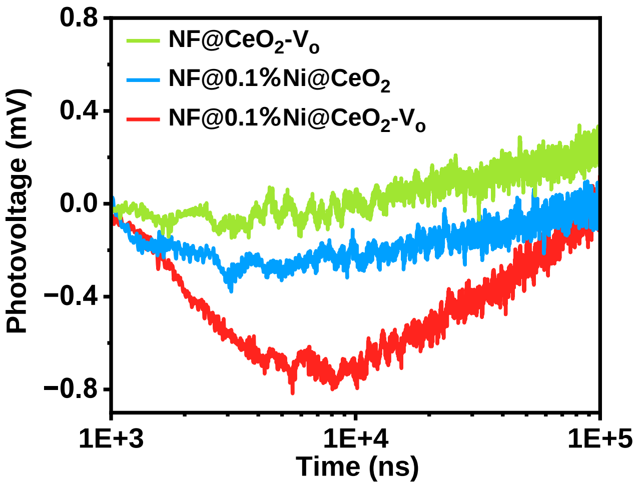


**Supplementary Fig. 50 |** Transient surface photovoltage of the catalysts, that is taking the logarithm of the abscissa of Fig. 4c.

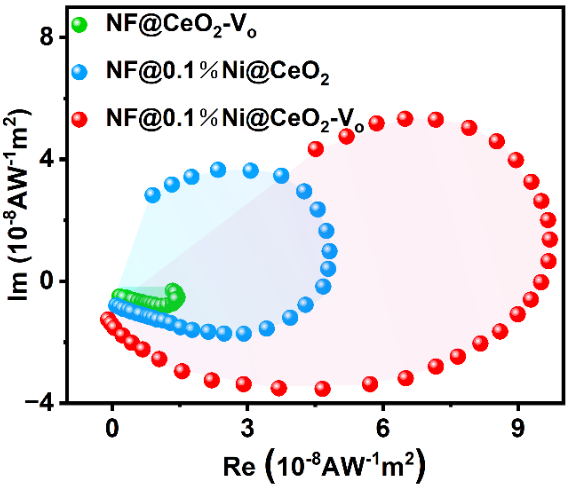


**Supplementary Fig. 51 |** IMPS spectra of different catalysts under the applied bias of 1.2 V. The charge transfer and relaxation are reflected by the upper quadrant semicircle, while the competition between interfacial charge transfer and complexation is represented by the lower quadrant semicircle.

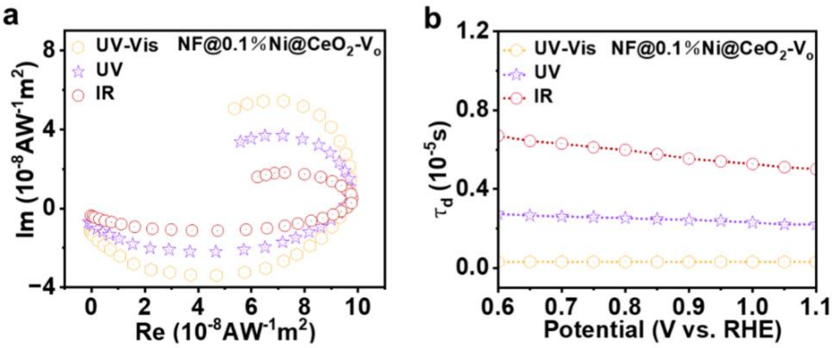


**Supplementary Fig. 52 | a, b** IMPS spectra at UV, UV-Vis, and IR wavelengths (**a**), and a plot of the average lifetime of photoinduced electrons ($\tau_d$) vs. potential (**b**) for NF@0.1%Ni@$CeO_2$-$V_o$.

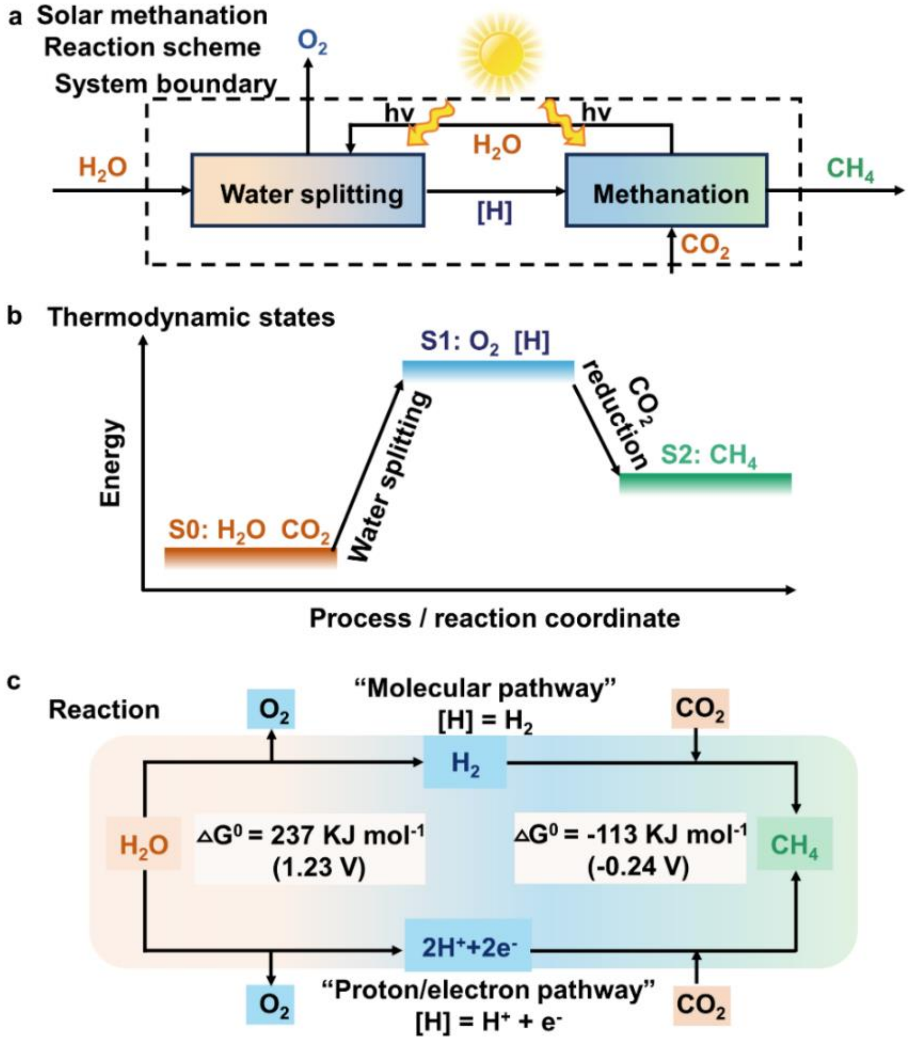


**Supplementary Fig. 53 | a-c** Schematic diagram of the solar methanation reaction scheme (**a**), thermodynamic states (**b**), and reaction pathways (**c**).

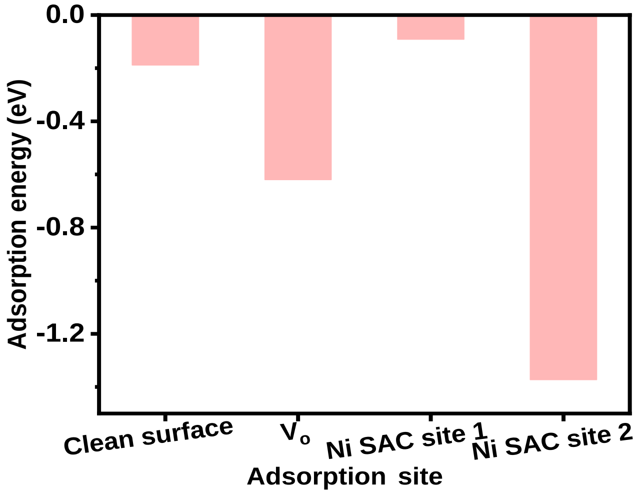


**Supplementary Fig. 54 |** Adsorption energies of $CO_2$ on clean surface of $CeO_2$ (111), $V_o$ site and Ni SAC site.

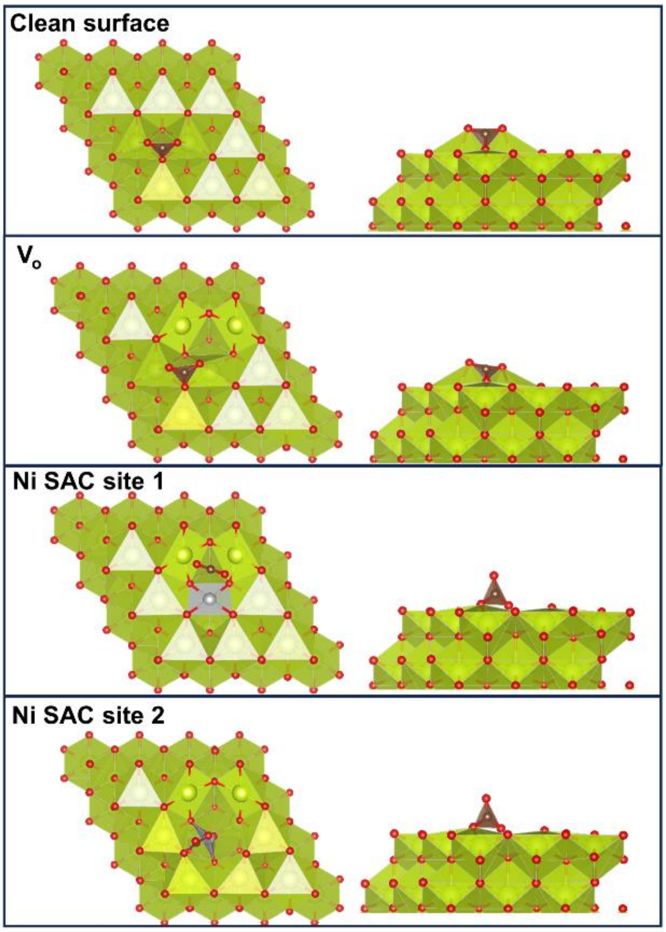


**Supplementary Fig. 55 |** Adsorption structure of $CO_2$ on clean surface of $CeO_2$ (111), $V_o$ site and Ni SAC site.

**Supplementary Discussion:** If $CO_2$ is adsorbed on the Ni single-atom via a C–Ni bond,

the adsorption energy is very low, making it ineffective. This is primarily attributed to the strong electrostatic repulsion between the Ni atom and the C atom. Therefore, the C atom in $CO_2$ can bond with lattice oxygen, causing a slight deviation of the Ni single atom from its initial position and forming a bond with the O atom in $CO_2$. Subsequently, this leads to a more efficient adsorption process.

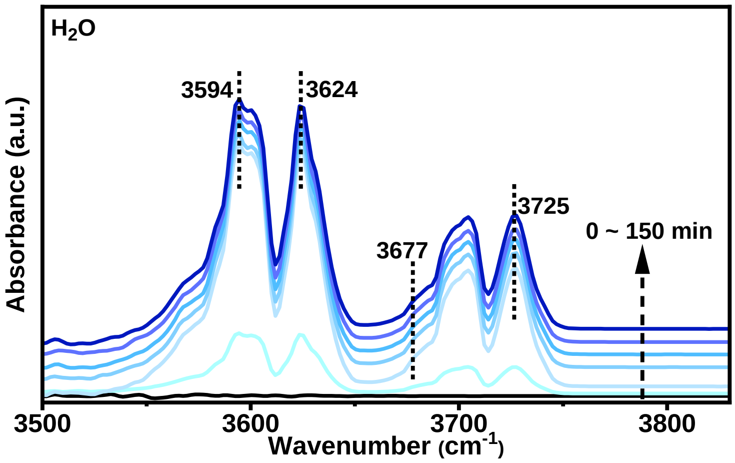


**Supplementary Fig. 56 |** In-situ DRIFTS spectra between 3500-3830 $cm^{-1}$ of $CO_2/H_2O$ on the NF@0.1%Ni@$CeO_2$-$V_o$ catalyst.

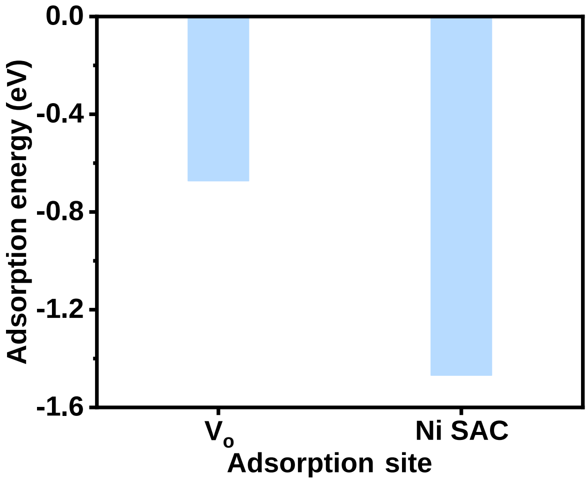


**Supplementary Fig. 57 |** Adsorption energies of $H_2O$ on $V_o$ and Ni SAC site.

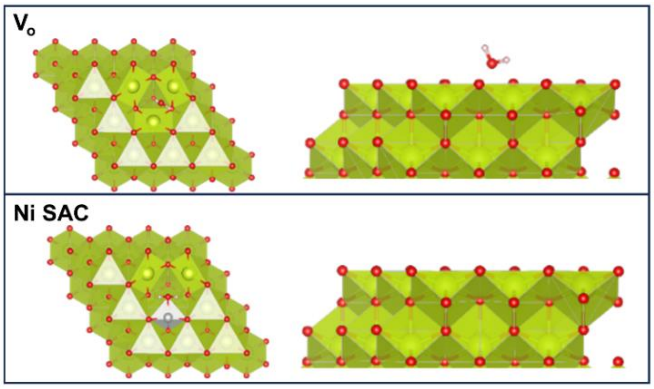


**Supplementary Fig. 58 |** Adsorption structure of $H_2O$ on $V_o$ site and Ni SAC site.

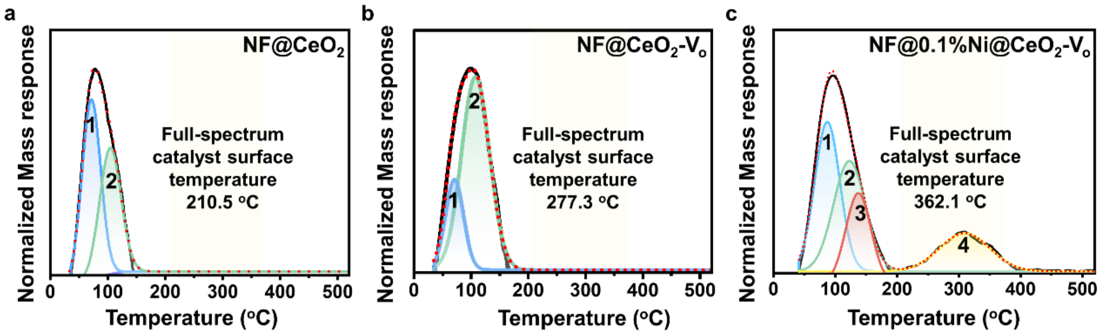


**Supplementary Fig. 59 | a, b, and c** TPD spectra from water adsorbed on NF@$CeO_2$ (**a**), NF@$CeO_2$-$V_o$ (**b**), NF@0.1%Ni@$CeO_2$-$V_o$ (**c**).

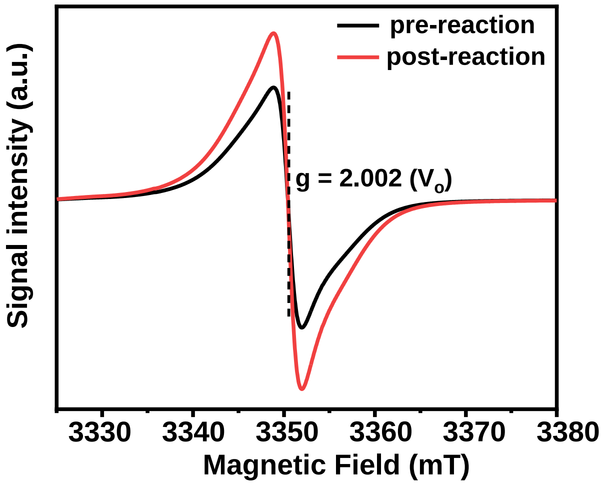


**Supplementary Fig. 60 |** EPR spectra of before and after the catalyst reaction.

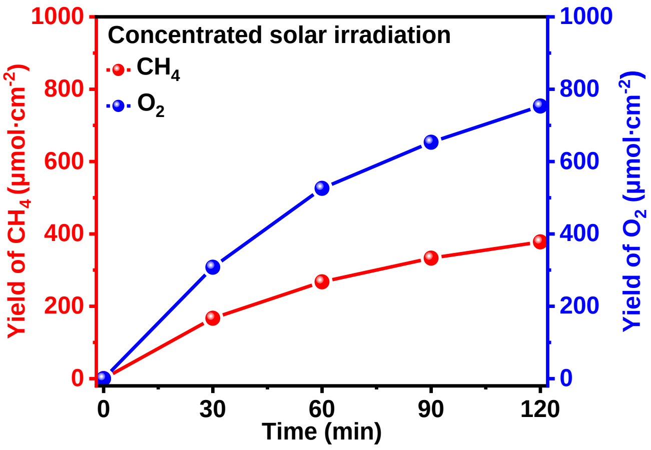


**Supplementary Fig. 61 |** The spectrum of $O_2$ yield for NF@0.1%Ni@$CeO_2$-$V_o$.

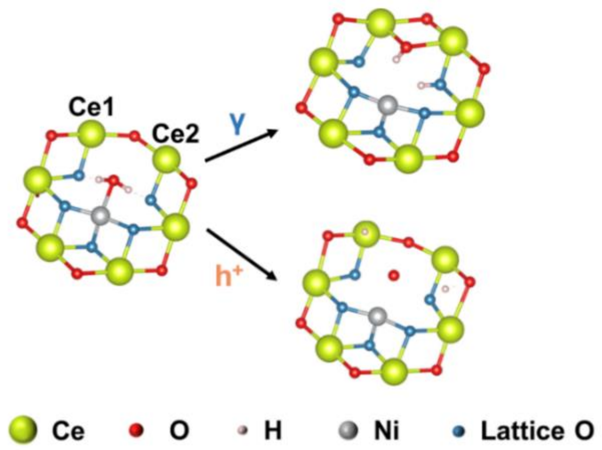


**Supplementary Fig. 62 |** Microstructure change during $H_2O$ dissociation via heat-induced and laser-induced reaction processes.

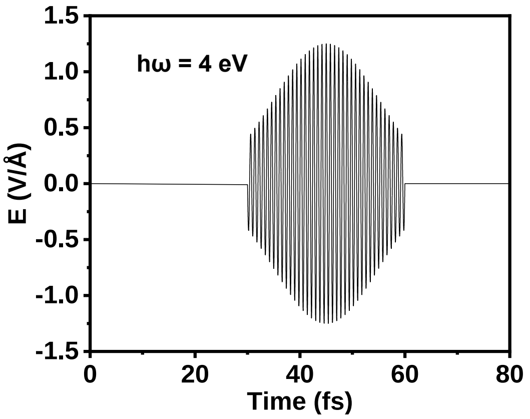


**Supplementary Fig. 63 |** Time-dependent electric field strength of the external laser field.

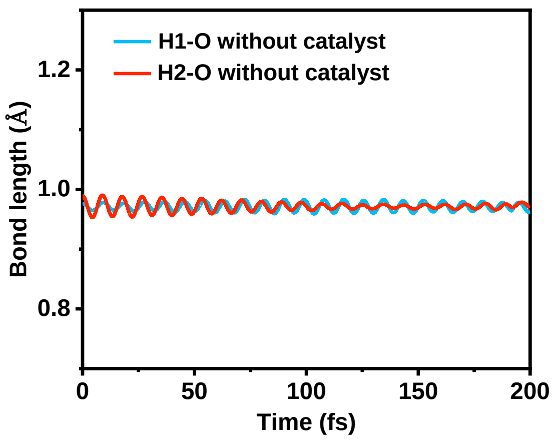


**Supplementary Fig. 64 |** Time-dependent H-O bond length of $H_2O$ stimulated by external laser field without catalysts.

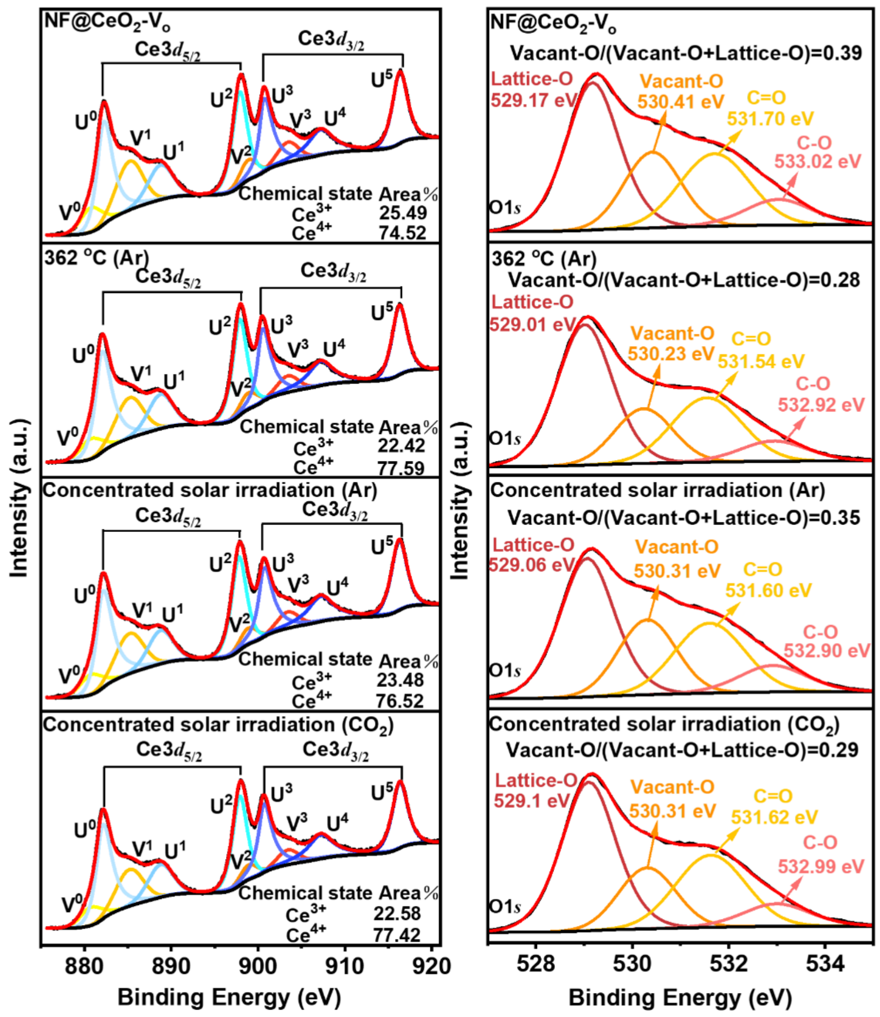


**Supplementary Fig. 65 |** High-resolution XPS spectra of the NF@$CeO_2$-$V_o$ catalyst under different conditions.

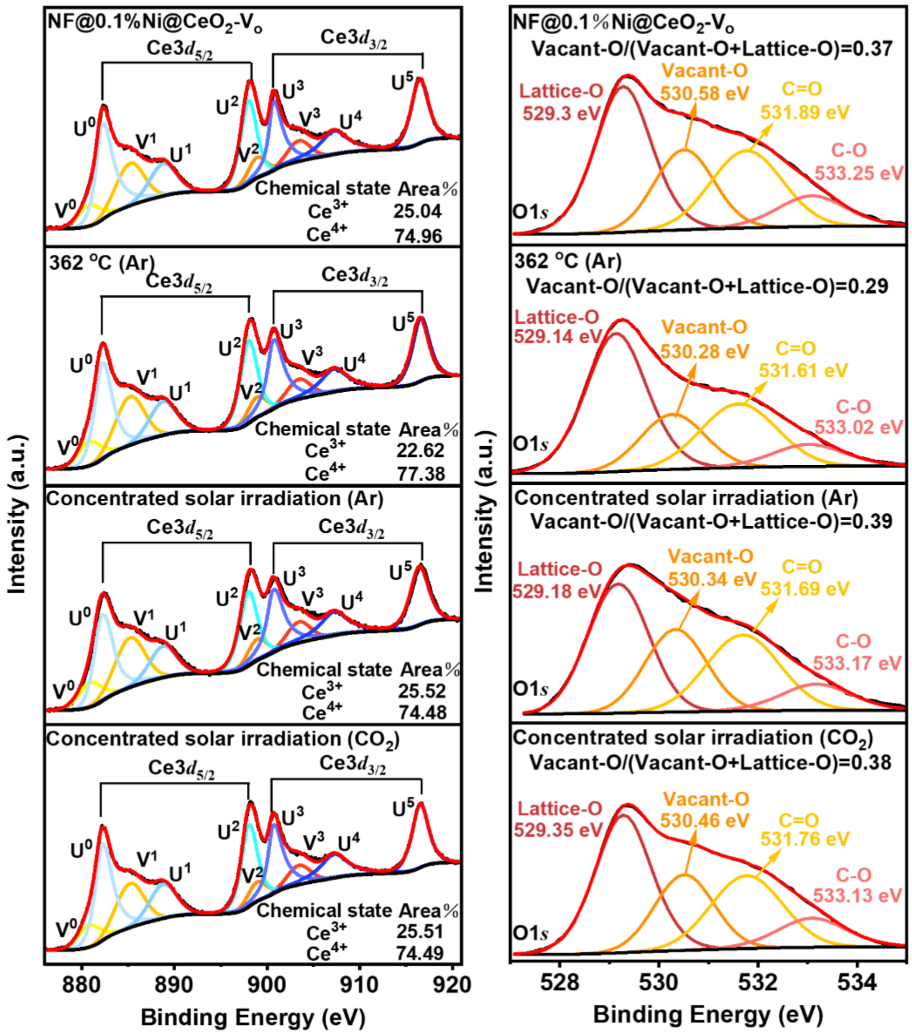


**Supplementary Fig. 66 |** High-resolution XPS spectra of the NF@0.1%Ni@$CeO_2$-$V_o$ catalyst under different conditions.

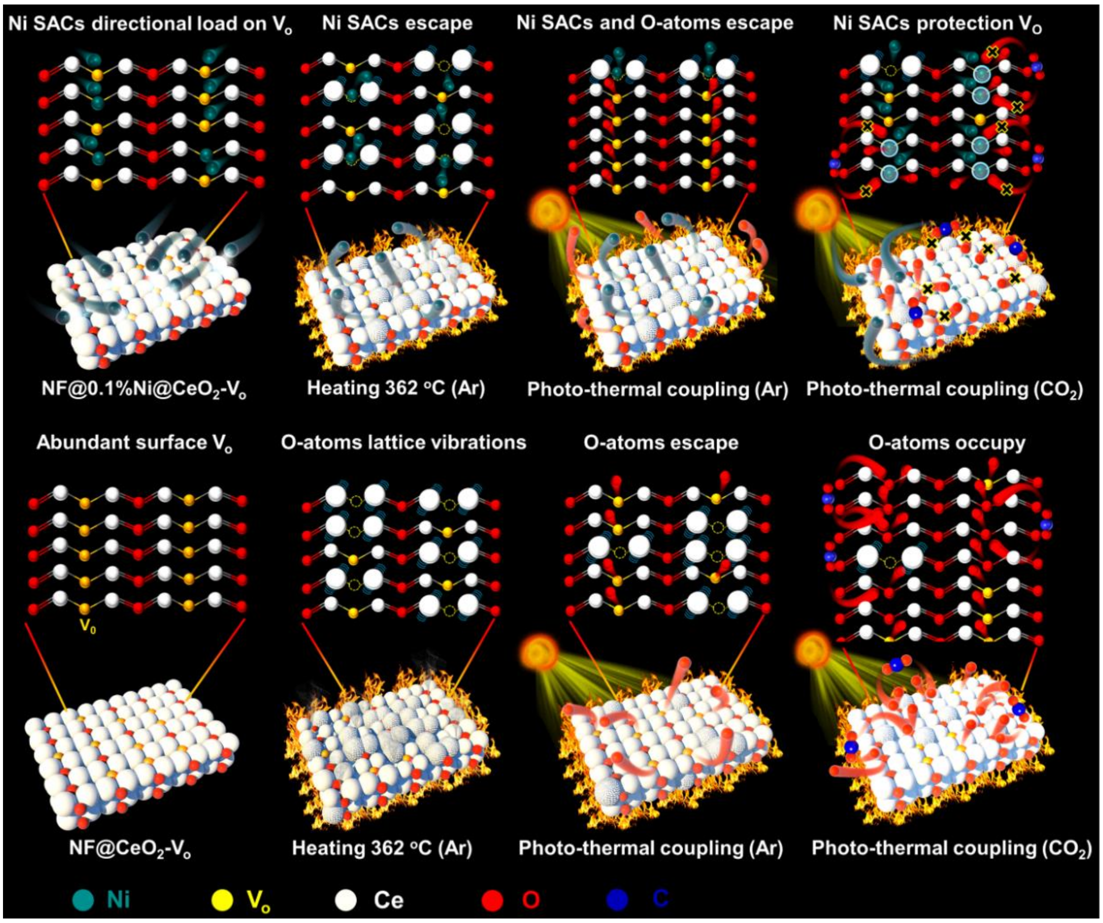


**Supplementary Fig. 67 |** Mechanistic diagram based on XPS results of the NF@$CeO_2$-$V_o$ and NF@0.1%Ni@$CeO_2$-$V_o$ catalysts under different conditions.

**Supplementary Discussion:** The results revealed the following findings: 1) the introduction of Ar upon heating (362 °C) without light irradiation results in a decrease in the $V_o$ content in the catalysts. This can be attributed to the high temperature promoting the diffusion rate of O ions within the $CeO_2$ lattice and facilitating the filling of $V_o$. Additionally, lattice vibrations may promote the adsorption and reaction of $O_2$ on the $CeO_2$ surface. 2) The introduction of Ar under concentrated solar irradiation enhances the $V_o$ content on the catalyst surface. This could be attributed to the injection of photo-generated electrons into the mid-gap state, which corresponds to the anti-bonding orbital of the Ni–O octahedron, facilitating the delocalisation of surface lattice O (Fig. 2b). 3) Following the photo-thermal coupled reduction of $CO_2$ with $H_2O$, the $V_o$ content in NF@$CeO_2$-$V_o$ considerably decreased. Conversely, the $V_o$ content in NF@0.1%Ni@$CeO_2$-$V_o$ remained almost unchanged. This suggests that the presence of single-atom Ni protects the $V_o$, preventing them from being filled.

**Supplementary Table 1 |** Textural parameters of as-synthesized samples.

| Sample | BET surface area [$m^2/g$][a] | Pore Volume [$cm^3/g$][b] | Pore size [nm][c] |
|---|---|---|---|
| NF@0.1%Ni@$CeO_2$ | 36.959 | 0.119157 | 15.0132 |
| NF@0.1%Ni@$CeO_2$-$V_o$ | 145.5063 | 0.387066 | 10.6815 |

[a] Surface area calculated by BET Surface Area.

[b] Single point total pore volume calculated by the nitrogen amount adsorbed.

[c] Average pore size calculated by BJH desorption average pore diameter (4V/A).

**Supplementary Table 2 |** Best-fitted EXAFS results of Ni[a].

| Sample | Shell | CN | R (Å) | $\sigma^{\wedge 2}$ ($10^{-2}$ $Å^2$) | $\Delta E_0$ (eV) | r-factor (%) |
|---|---|---|---|---|---|---|
| Ni foil | Ni–Ni | 12 | 2.48 | 0.4 | 5.4 | 0.07 |
| NF@0.1%Ni@$CeO_2$ | Ni–O | 5.1 | 2.04 | 0.7 | -8.8 | 0.2 |
| NF@0.1%Ni@$CeO_2$-$V_o$ | Ni–O | 4.3 | 2.03 | 0.7 | -8.5 | 0.3 |

[a] CN is the coordination number for the absorber-backscatterer pair, R is the average absorber-backscatterer distance, $\sigma^2$ is the Debye-Waller factor, and $\Delta E_0$ is the inner potential correction. The accuracies of the above parameters are estimated as CN, ±20%; R, ±1%; $\sigma^2$, ±20%; $\Delta E_0$, ±20%. The data range used for data fitting in k-space (Δk) and R-space (ΔR) is 3.0-12.6 $Å^{-1}$ and 1.0-2.0 Å, respectively.

**Supplementary Table 3 |** Chemical bond lengths of the NF@0.1%Ni@$CeO_2$ and NF@0.1%Ni@$CeO_2$-$V_o$ catalysts obtained by DFT simulations.

| | Chemical bond | Ni–O1 | Ni–O2 | Ni–O3 | Ni–O4 | Ni–O5 | Ni–O6 |
|---|---|---|---|---|---|---|---|
| NF@0.1%Ni@$CeO_2$ | Bond length (Å) | 2.09 | 2.03 | 2.09 | 2.07 | 2.01 | 2.01 |
| NF@0.1%Ni@$CeO_2$-$V_o$ | | - | 1.96 | 2.11 | 2.11 | 1.97 | 2.69 |

**Supplementary Table 4 |** Best-fitted EXAFS results of Ce.

| Sample | Shell | CN | R (Å) | $\sigma^{\wedge 2}$ ($10^{-2}$ Å$^2$) | $\Delta E_0$ (eV) | r-factor (%) |
|---|---|---|---|---|---|---|
| $CeO_2$ | Ce–O | 8.0* | 2.29 | 1.0 | 5.5 | 0.3 |
| NF@0.1%Ni@$CeO_2$ | Ce–O | 7.1 | 2.31 | 0.4 | 3.7 | 0.1 |
| NF@0.1%Ni@$CeO_2$-$V_o$ | Ce–O | 6.1 | 2.28 | 0.8 | 2.8 | 0.3 |

[a] CN is the coordination number for the absorber-backscatterer pair, R is the average absorber-backscatterer distance, $\sigma^2$ is the Debye-Waller factor, and $\Delta E_0$ is the inner potential correction. $^*S_0^2$ was fixed to 0.75 as determined from the $CeO_2$ fitting. The accuracies of the above parameters are estimated as CN, ±20%; R, ±1%; $\sigma^2$, ±20%; $\Delta E_0$, ±20%. The data range used for data fitting in k-space (Δk) and R-space (ΔR) is 3.0-10.1 Å$^{-1}$ and 1.4-2.3 Å, respectively.

**Supplementary Table 5 |** Comparison of energy conversion efficiencies of photo and photothermal catalysis.

| Catalysts | Light Intensity (mW cm$^{-2}$) | Reaction medium | Reduction products | Catalyst dosage (mg) | System temperature (K) | Yield | $\eta_{STC}$ (%) |
|---|---|---|---|---|---|---|---|
| NF@0.1%Ni@$CeO_2$-$V_o$ (This work) | 4200 | $CO_2$ and $H_2O$ vapour | $CH_4$ | 1 | 635.15 | 192.75 μmol cm$^{-2}$·h$^{-1}$ | 1.14 |
| $WO_3$ | 4000 | $CO_2$ and $H_2O$ vapour | CO and $CH_4$ | 1 | 511.15 | 26.51 and 3.36 μmol·cm$^{-2}$·h$^{-1}$ | 0.073 |
| $ZrO_2$ | 4000 | $CO_2$ and $H_2O$ vapour | CO and $CH_4$ | 1 | 529.15 | 20.63 and 7.36 μmol·cm$^{-2}$·h$^{-1}$ | 0.086 |
| $TiO_2$ | 4000 | $CO_2$ and $H_2O$ vapour | CO and $CH_4$ | 1 | 485.15 | 15.42 and 2.65 μmol·cm$^{-2}$·h$^{-1}$ | 0.047 |
| Au-Mt-H6[1] | 720 | $CO_2$ and $H_2O$ vapour | CO and $CH_4$ | 10 | 454.15 | 0.14 and 0.17 μmol·m$^{-2}$ | 0.000037 |
| TF@TNT/0.4$CoO_x$-0.1CuO[2] | 4266 | $CO_2$ and $H_2O$ | CO and $CH_4$ | 1 | 573.15 | 8.45 and 58.2 μmol·cm$^{-2}$·h$^{-1}$ | 0.35 |

| | | | | | | | |
|---|---|---|---|---|---|---|---|
| | | vapour | | | | | |
| $Bi_2S_3@In_2S_3$[3] | 1150 | $CO_2$ and $H_2O$ vapour | CO and $C_2H_4$ | 5 | 513.15 | 1.95 and 11.81 $\mu mol\cdot g^{-1}\cdot h^{-1}$ | 0.00033 |
| Pt/ZnO[4] | 4200 | $CO_2$ and $H_2O$ vapour | CO and $CH_4$ | 20 | 540.15 | 115.32 and 8 $\mu mol\cdot g^{-1}\cdot h^{-1}$ | 0.264 |
| $Au_x$/TJU-16[5] | 300 W Xe lamp | $CO_2$ and $H_2O$ vapour | CO and $CH_4$ | 40 | / | 10.1 and 2.5 $\mu mol\cdot g^{-1}\cdot h^{-1}$ | 0.034 |
| $Au@Cr_2O_3$[6] | 1600 | $CO_2$ and $H_2O$ vapour | CO and $H_2$ | 1000 | / | 0.074 and 0.013 $mol\cdot g^{-1}\cdot h^{-1}$ | 0.89 |
| $CuO_x/TiO_2$[7] | 300 W Xe lamp | $CO_2$ and $H_2O$ vapour | CO and $CH_4$ | 50 | / | 0.32 and 2.29 $\mu mol\cdot g^{-1}\cdot h^{-1}$ | 0.0372 |
| $Sn/In_2O_3$[8] | 300 W Xe lamp | $CO_2$ and $H_2O$ vapour | CO | 50 | / | 17.9 $\mu mol\cdot g^{-1}\cdot h^{-1}$ | 0.05 |
| Rh/Al nanoantenna[9] | 11300 | $CO_2$ and $H_2$ | $CH_4$ and $H_2O$ | 20 | 973.15 | 550 $mmol\cdot g^{-1}\cdot h^{-1}$ | 0.0011 |
| $Co/Al_2O_3$[10] | 3295 | $CO_2$ and $H_2$ | $CH_4$ | / | 803.15 | 6036 $\mu mol\cdot g^{-1}\cdot h^{-1}$ | 0.0002 |

**Supplementary Table 6 |** Selectivity of $CH_4$ under photothermal conditions with different catalysts.

| Sample | Selectivity (%) |
|---|---|
| NF@0.1%Ni@$CeO_2$ (This work) | 78 |
| NF@0.1%Ni@$CeO_2$-$V_o$ (This work) | ≈100 |
| $Ni@CeO_2$[11] | 70 |
| $Ni_{75}Pt_{25}/Al_2O_3$[12] | 48.2 |
| $(SnS_2)_1$/3DOM-$SrTiO_3$[13] | 61.6 |
| 1.0Pt/$In_2O_3$[14] | 51.9 |
| Pt/D-$TiO_{2-x}$[15] | 87.5 |
| BW[16] | 50.3 |
| 10%Ni-$TiO_2$[17] | 83 |

| | | |
|---|---|---|
| 10%Ni-$TiO_2$-anatase[18] | 79 | |
| LDH-S2[19] | 83.07 | |
| $Pt_2/P_{25}$[20] | 73.9 | |
| F-MSCS[21] | 85.8 | 85.8 |

**Supplementary Table 7 |** Best-fitted EXAFS results of Ni.

| Sample | Shell | CN | R (Å) | $\sigma^{\wedge 2}$ ($10^{-2}$ Å$^2$) | $\Delta E_0$ (eV) | r-factor (%) |
|---|---|---|---|---|---|---|
| Ni foil | Ni–Ni | 12 | 2.48 | 0.4 | 5.4 | 0.07 |
| NF@0.1%Ni@$CeO_2$-$V_o$ | Ni–O | 4.3 | 2.03 | 0.7 | -8.5 | 0.3 |
| NF@0.1%Ni@$CeO_2$-$V_o$ (After reaction) | Ni–O | 4.4 | 2.04 | 0.7 | -8.1 | 0.3 |

[a] CN is the coordination number for the absorber-backscatterer pair, R is the average absorber-backscatterer distance, $\sigma^2$ is the Debye-Waller factor, and $\Delta E_0$ is the inner potential correction. The accuracies of the above parameters are estimated as CN, ±20%; R, ±1%; $\sigma^2$, ±20%; $\Delta E_0$, ±20%. The data range used for data fitting in k-space (Δk) and R-space (ΔR) is 3.0-12.6 Å$^{-1}$ and 1.0-2.0 Å, respectively.

**Supplementary Table 8 |** Best-fitted EXAFS results of Ce.

| Sample | Shell | CN | R (Å) | $\sigma^{\wedge 2}$ ($10^{-2}$ Å$^2$) | $\Delta E_0$ (eV) | r-factor (%) |
|---|---|---|---|---|---|---|
| $CeO_2$ | Ce–O | 8.0* | 2.29 | 1.0 | 5.5 | 0.3 |
| NF@0.1%Ni@$CeO_2$-$V_o$ | Ce–O | 6.1 | 2.28 | 0.8 | 2.8 | 0.3 |
| NF@0.1%Ni@$CeO_2$-$V_o$ (After reaction) | Ce–O | 6.3 | 2.26 | 0.8 | 0.2 | 0.3 |

[a] CN is the coordination number for the absorber-backscatterer pair, R is the average absorber-backscatterer distance, $\sigma^2$ is the Debye-Waller factor, and $\Delta E_0$ is the inner potential correction. *$S_0^2$ was fixed to 0.75 as determined from the $CeO_2$ fitting. The accuracies of the above parameters are estimated as CN, ±20%; R, ±1%; $\sigma^2$, ±20%; $\Delta E_0$,

±20%. The data range used for data fitting in k-space (Δk) and R-space (ΔR) is 3.0-10.1 $Å^{-1}$ and 1.4-2.3 Å, respectively.

**Supplementary Table 9 |** Parameters for $CH_4$ production under concentrated solar irradiation and non-concentrated solar irradiation.

| 1000/T ($K^{-1}$) | $\ln k_{CH4}$ yield ($\mu mol/cm^2/h$) | 1000/T ($K^{-1}$) | $\ln k_{CH4}$ yield ($\mu mol/cm^2/h$) |
|---|---|---|---|
| 1.57 | 5.89 | 2.62 | 1.59 |
| 1.68 | 5.76 | 2.7 | 1.49 |
| 1.81 | 5.68 | 2.8 | 1.119 |
| 1.93 | 5.6 | 2.92 | 0.89 |
| 2.06 | 5.56 | 3.02 | 0.66 |

**Supplementary Table 10 |** Experimental details parameters.

| Cooling circulating water temperature (°C) | Temperature of the inner chamber (°C) [a] | Water circulation flow rate (mL/min) |
|---|---|---|
| 0 | 50 | 600 |
| 0 | 100 | 240 |
| 30 | 150 | 600 |
| 30 | 200 | 240 |
| / | 235 | / |

[a] Temperature tested by thermocouple.

**Supplementary Table 11 |** Variation of CO yields with temperature.

| Temperature (°C) | CO Yield ($\mu mol/cm^2/h$) | $CH_4$ Yield ($\mu mol/cm^2/h$) |
|---|---|---|
| 50 | 0.15 | 59.34 |
| 100 | 0.21 | 79.64 |
| 150 | 0.32 | 117.41 |
| 200 | 0.48 | 140.32 |

**Supplementary Table 12 |** Emission lifetimes ($\tau_1$, $\tau_2$, $\tau$) of the samples.

| Sample | $\tau_1$ (μs) | $A_1$ (%) | $\tau_2$ (μs) | $A_2$ (%) | $\tau$ (μs) |
|---|---|---|---|---|---|
| NF@$CeO_2$-$V_o$ | 1.21 | 1193.38 | 113.2 | 1865.46 | 112.43 |
| NF@0.1%Ni@$CeO_2$ | 1.27 | 1284.59 | 114.8 | 1777.45 | 113.91 |
| NF@0.1%Ni@$CeO_2$-$V_o$ | 1.28 | 1318.31 | 114.2 | 1731.95 | 114.42 |

**Supplementary Table 13 |** Lifetimes ($\tau_1$, $\tau_2$, $\tau$) of carriers tested by TPV.

| Sample | $\tau_1$ (ns) | $A_1$ (%) | $\tau_2$ (ns) | $A_2$ (%) | $\tau$ (ns) |
|---|---|---|---|---|---|
| NF@$CeO_2$-$V_o$ | 89782.47 | 7 | 89749.3 | 4.29 | 89769.87 |
| NF@0.1%Ni@$CeO_2$ | 94157.29 | 10.76 | 9152.77 | 2.82 | 92045.49 |
| NF@0.1%Ni@$CeO_2$-$V_o$ | 112499.54 | 3.79 | 9926.08 | 1.04 | 110074.79 |

**Supplementary Table 14 |** Elemental compositions from XPS.

| Sample | Lattice-O[a] | Vacant-O[a] |
|---|---|---|
| NF@$CeO_2$-$V_o$ | 39.61 | 25.02 |
| NF@$CeO_2$-$V_o$[b] | 46.96 | 18.58 |
| NF@$CeO_2$-$V_o$[c] | 41.73 | 22.83 |
| NF@$CeO_2$-$V_o$[d] | 45.56 | 18.83 |
| NF@0.1%Ni@$CeO_2$-$V_o$ | 40.88 | 23.82 |
| NF@0.1%Ni@$CeO_2$-$V_o$[b] | 46.55 | 18.84 |
| NF@0.1%Ni@$CeO_2$-$V_o$[c] | 38.56 | 24.64 |
| NF@0.1%Ni@$CeO_2$-$V_o$[d] | 38.40 | 24.56 |

[a] Calculated from XPS results.

[b] Catalyst was calcined at 362 °C under an Ar atmosphere for 2 h.

[c] Catalyst was irradiated with a concentrated solar (light) reactor under an Ar atmosphere for 2 h.

[d] Catalyst was irradiated with a concentrated solar (light) reactor under a $CO_2$ atmosphere for 2 h.

**Supplementary Table 15 |** Nominal and measured Ni contents in NF@0.1%Ni@$CeO_2$ and NF@0.1%Ni@$CeO_2$-$V_o$ catalysts.

| Sample | Nominal Ni content (wt%) | Measured Ni content[a] (wt%) |
|---|---|---|
| NF@0.1%Ni@$CeO_2$ | 0.1 | 0.13 |
| NF@0.1%Ni@$CeO_2$-$V_o$ | 0.1 | 0.12 |

[a] The Ni content was measured by ICP-MS.

## Supplementary References


1 Cai, S., Chen, J., Li, Q. & Jia, H. Enhanced photocatalytic $CO_2$ reduction with photothermal effect by cooperative effect of oxygen vacancy and Au cocatalyst. *ACS Appl. Mater. Interfaces* **13**, 14221-14229 (2021).

2 Xu, L. *et al.* Strong photo-thermal coupling effect boosts $CO_2$ reduction into $CH_4$ in a concentrated solar reactor. *Chem. Eng. J.* **468**, 143831 (2023).

3 Yan, K. *et al.* Highly selective ethylene production from solar-driven $CO_2$ reduction on the $Bi_2S_3$@$In_2S_3$ catalyst with In–$S_V$–Bi active sites. *ACS Catal.* **13**, 2302-2312 (2023).

4 Wu, M. *et al.* Photothermal coupling effect boosts the conversion of $CO_2$ to solar fuel over Pt/ZnO photocatalyst in a concentrated solar reactor. *Ind. Eng. Chem. Res.* **62**, 9463-9473 (2023).

5 Chen, R., Gao, G. & Luo, J. A water-stable organolead iodide material for overall photocatalytic $CO_2$ reduction. *Nano Res.* **15**, 10084-10089 (2022).

6 Rashid, R. T. *et al.* Tunable green syngas generation from $CO_2$ and $H_2O$ with sunlight as the only energy input. *PNAS* **119**, e2121174119 (2022).

7 Fang, F. *et al.* $TiO_2$ Facet-dependent reconstruction and photocatalysis of $CuO_x/TiO_2$ photocatalysts in $CO_2$ photoreduction. *Appl. Surf. Sci.* **564**, 150407 (2021).

8 Cao, G. *et al.* Plasmon enhanced $Sn:In_2O_3$/attapulgite S-scheme heterojunction for efficient photothermal reduction of $CO_2$. *Colloids Surf. A Physicochem. Eng. Aspects* **656**, 130398 (2023).

9 Fu, G. *et al.* Rh/Al nanoantenna photothermal catalyst for wide-spectrum solar-driven $CO_2$ methanation with nearly 100% selectivity. *Nano Lett.* **21**, 8824-8830 (2021).

10 Chen, X. *et al.* MOF-templated preparation of highly dispersed $Co/Al_2O_3$ composite as the photothermal catalyst with high solar-to-fuel efficiency for $CO_2$ methanation. *ACS Appl. Mater. Interfaces* **12**, 39304-39317 (2020).

11 Jia, Z. *et al.* Selective photothermal reduction of $CO_2$ to CO over Ni-nanoparticle/N-doped $CeO_2$ nanocomposite catalysts. *ACS Appl. Nano Mater.* **4**, 10485-10494 (2021).

12 Kikkawa, S., Teramura, K., Asakura, H., Hosokawa, S. & Tanaka, T. Isolated platinum atoms in $Ni/\gamma\text{-}Al_2O_3$ for selective hydrogenation of $CO_2$ toward $CH_4$. *J. Phys. Chem. C* **123**, 23446-23454 (2019).

13 He, W. *et al.* Z-scheme heterojunction of $SnS_2$-decorated 3DOM-$SrTiO_3$ for selectively photocatalytic $CO_2$ reduction into $CH_4$. *Chin. Chem. Lett.* **31**, 2774-2778 (2020).

14 Wang, Y., Zhao, J., Li, Y. & Wang, C. Selective photocatalytic $CO_2$ reduction to $CH_4$ over $Pt/In_2O_3$: Significant role of hydrogen adatom. *Appl. Catal. B Environ.* **226**, 544-553 (2018).

15 Yu, F. *et al.* Revisiting $Pt/TiO_2$ photocatalysts for thermally assisted photocatalytic reduction of $CO_2$. *Nanoscale* **12**, 7000-7010 (2020).

16 Li, Y.-Y. *et al.* Selective photocatalytic reduction of $CO_2$ to $CH_4$ modulated by chloride modification on $Bi_2WO_6$ nanosheets. *ACS Appl. Mater. Interfaces* **12**, 54507-54516 (2020).

17 Makdee, A., Kidkhunthod, P., Poo-arporn, Y. & Chanapattharapol, K. C. Enhanced $CH_4$ selectivity for $CO_2$ methanation over Ni-$TiO_2$ by addition of Zr promoter. *J. Environ. Chem. Eng.* **10**, 107710 (2022).

18 Messou, D. *et al.* Origin of the synergistic effect between $TiO_2$ crystalline phases in the $Ni/TiO_2$-catalyzed $CO_2$ methanation reaction. *J. Catal.* **398**, 14-28 (2021).

19 Tan, L., Sun, X., Bai, S., Song, Z. & Song, Y. F. Dual engineering of lattice strain and valence state of NiAl-LDHs for photoreduction of $CO_2$ to highly selective $CH_4$. *Small* **19**,

202205770 (2023).

20 Wu, X. *et al.* Multifunctional photocatalysts of Pt-decorated 3DOM perovskite-type $SrTiO_3$ with enhanced $CO_2$ adsorption and photoelectron enrichment for selective $CO_2$ reduction with $H_2O$ to $CH_4$. *J. Catal.* **377**, 309-321 (2019).

21 Xing, M. *et al.* Modulation of the reduction potential of $TiO_{2-x}$ by fluorination for efficient and selective $CH_4$ generation from $CO_2$ photoreduction. *Nano Lett.* **18**, 3384-3390 (2018).
